# The Multiple States of Environmental DNA and What Is Known about Their Persistence in Aquatic Environments

**DOI:** 10.1021/acs.est.1c07638

**Published:** 2022-04-18

**Authors:** Quentin Mauvisseau, Lynsey R. Harper, Michael Sander, Robert H. Hanner, Hannah Kleyer, Kristy Deiner

**Affiliations:** † Natural History Museum, University of Oslo, Sars’ gate 1, 0562 Oslo, Norway; ‡ Nature Metrics Ltd, CABI Site, Bakeham Lane, Egham, Surrey TW20 9TY, United Kingdom; § Department of Environmental Systems Science, ETH Zurich, Universitätstrasse 16, CH-8092 Zurich, Switzerland; ∥ Department of Integrative Biology, University of Guelph, 50 Stone Road East, Guelph, Ontario N1G 2W1, Canada

**Keywords:** environmental DNA, states, persistence, aquatic environments

## Abstract

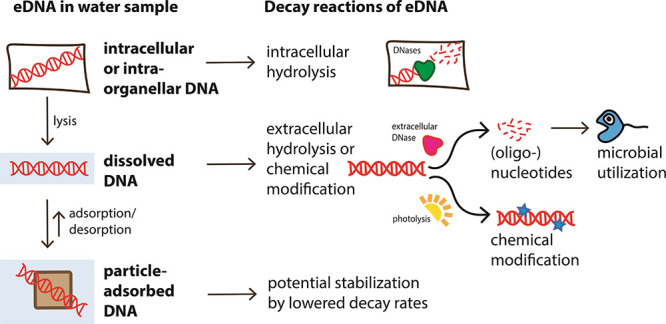

Increased use of environmental DNA (eDNA) analysis for indirect species detection has spurred the need to understand eDNA persistence in the environment. Understanding the persistence of eDNA is complex because it exists in a mixture of different states (e.g., dissolved, particle adsorbed, intracellular, and intraorganellar), and each state is expected to have a specific decay rate that depends on environmental parameters. Thus, improving knowledge about eDNA conversion rates between states and the reactions that degrade eDNA in different states is needed. Here, we focus on eukaryotic extraorganismal eDNA, outline how water chemistry and suspended mineral particles likely affect conversion among each eDNA state, and indicate how environmental parameters affect persistence of states in the water column. On the basis of deducing these controlling parameters, we synthesized the eDNA literature to assess whether we could already derive a general understanding of eDNA states persisting in the environment. However, we found that these parameters are often not being measured or reported when measured, and in many cases very few experimental data exist from which to draw conclusions. Therefore, further study of how environmental parameters affect eDNA state conversion and eDNA decay in aquatic environments is needed. We recommend analytic controls that can be used during the processing of water to assess potential losses of different eDNA states if all were present in a water sample, and we outline future experimental work that would help determine the dominant eDNA states in water.

## Introduction

Over the past decade, the use of environmental DNA (eDNA) based detection to monitor aquatic biodiversity in both marine and freshwater systems has rapidly increased.^1^ eDNA refers to the total pool of DNA isolated from the environment and is composed from both organismal (whole individuals that were probably alive at the time of sampling) and extraorganismal DNA (material shed from organisms, or biologically active propagules).^2^ Production sources and persistence state of extraorganismal DNA can differ and vary depending on the taxon and species and are likely to affect eDNA detection sensitivity.^3^ However, the reproducibility of eDNA surveys relies on the assumption that the DNA detected provides an accurate measure of presence of the local community or targeted species at the respective point in time and space.^4,5^ Many conservation and management strategies have now adopted eDNA-based surveys^6,7^ as this method allows species to be identified and monitored without physical observation.^8^ It is therefore urgent to understand the various processes that influence eDNA persistence in aquatic systems so that accurate inferences of a species’ presence can be made from the detection of its eDNA. Indeed, previous studies highlighted that eDNA detectability or stability can vary in systems depending on many parameters, including species-specific eDNA shedding rates, seasonality, and environmental conditions.^9−11^ When organisms shed DNA into the water column, this gives rise to extraorganismal eDNA (i.e., DNA no longer associated with its organism of origin) and can take the form of at least four states:^2,12^ (i) dissolved DNA, (ii) DNA bound to the surfaces of suspended particles,^5,9,12^ and DNA still encapsulated in either (iii) a cell or (iv) an organelle.^13^ What we currently lack is a robust understanding of how water chemistry and other environmental parameters affect which eDNA state (states) predominates (predominate) in specific aquatic environments and how they persist.

The state of the art is to extract eDNA from water and target a single species or whole communities of species using a set of primers and polymerase chain reaction (PCR).^14^ However, the presence of eDNA in different states has implications for data interpretation, as detection of species might be influenced by the “detectability” of a specific state that is the result of both the environmental parameters determining the state and the analytical workflow (i.e., preservation, capture, extraction, and detection methods) used to isolate the eDNA from the water column. Consequently, the relative distribution of eDNA among the different states could affect the probability of detection for a targeted species’ DNA. Therefore, the currently unknown stabilities of eDNA in different states combined with the lack of information on which eDNA states are being detected create a large uncertainty for the spatial and temporal inferences that can be made from extraorganismal eDNA detection.^5,15^ To reduce this uncertainty, we require a better understanding of the states that eDNA assumes, the processes converting eDNA between different states, and the variations in state-dependent eDNA decay rates. Indeed, variations of decay rates cannot be fully understood without knowing both the molecular state of eDNA in water and environmental factors.^11^

In this critical review, we describe four principal states of eDNA that are likely in aquatic environments. On the basis of the presumed chemical behavior of each state, we discuss how environmental parameters, such as temperature, pH, and suspended particles, may influence the conversion of eDNA between states.^16^ We briefly review what is known about DNA decay, covered in detail elsewhere,^3,12,13^ and summarize what has been observed from experimental studies on eDNA decay in relation to the environmental parameters of temperature and pH. We then present the results of a literature search to ascertain what states of eDNA are likely being detected using single-species eDNA assays. Lastly, we outline a number of analytic controls, which, if used, will help to assess the loss of specific states from aquatic samples and allow for *post hoc* observations about the state(s) contributing to species detection. We close with suggestions for future research that would help to fill knowledge gaps regarding the space and time inference that can be made from extraorganismal eDNA species detections.

## Different States of eDNA

**Figure 1:**
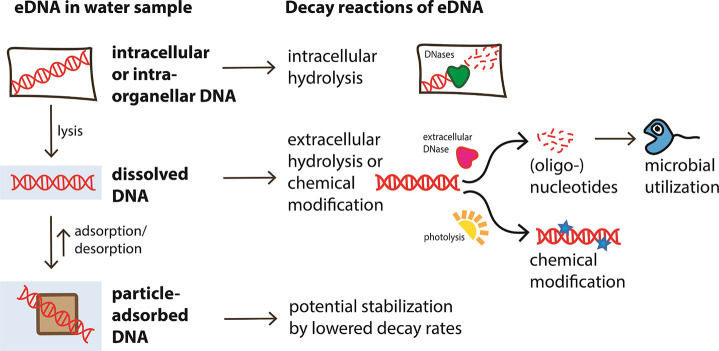
Summary of eDNA states, the processes that convert eDNA between states (cell lysis and adsorption/desorption), and the chemical reactions (intra- and extracellular breakdown, microbial utilization, or stabilization) that degrade or alter eDNA in different states making it inaccessible to capture and detection.

Environmental DNA can be present in four principal states described in Figure 1. Here we focus on eukaryotic extraorganismal eDNA,^2^ which is commonly analyzed to make accurate inferences as to whether or not a targeted species (usually of conservation or management concern) or community was present at time of sampling.^17^ Additionally, we focus on eDNA states at the cellular level and below because all eukaryotic life forms have cells as a basic unit encapsulating DNA. We recognize that extraorganismal eDNA may also originate from even more complex structures such as tissues and gametes, but variations in these structures are complex across eukaryotes and are beyond the scope of what we address in this critical review. However, this variation in tissues and other structures is likely a main factor that contributes to species-specific rates of DNA degradation and persistence.

The simplest form in which extraorganismal eDNA is present is a purely dissolved state. DNA is a highly water-soluble polyelectrolyte due to the negatively charged phosphodiester groups in the DNA backbone. However, dissolved DNA interacts with and may adsorb to the surfaces of mineral and organic particles and colloids suspended in the water. Particle-adsorbed DNA is therefore a second state. Existing literature on DNA adsorption^18−25^ suggests that DNA–particle interactions are mainly controlled by electrostatics (which may be either attractive or repulsive for positively and negatively charged particle surfaces, respectively) as well as inner sphere complex formation on some mineral surfaces.^26−31^ The bases connected to deoxyribose (i.e., cytosine, adenine, guanine, and thymine) likely only play a small, modulating role on DNA adsorption processes (i.e., these bases are involved in H bonding between the two complementary DNA strands). DNA can also remain associated with cells that are shed by organisms into the water, either as intracellular DNA (third state) or as intraorganellar DNA (fourth state) such as skin cells from mucus or cells from the intestinal tract during defecation. The types of cells shed from any organism and their source remain mostly undescribed, but recent advances using mRNA typing may allow us to gain a better understanding of the sources and types of cells that make up eDNA;^3^ for instance, intraorganellar DNA may be present in mitochondria and chloroplasts. In fact, many extraorganismal eDNA studies target genes found in organelles due to their high copy number per cell which should increase the probability of eDNA detection. Moreover, multicopy nuclear DNA has also been found to be a sensitive eDNA marker^32,33^ and indicative of reproduction and age class when combined with a mitochondrial DNA marker.^12,34^

## State Conversion Processes

### Cell and Organelle Lysis

The sources of extracellular DNA in water samples are cells that cover a broad range of properties and characteristics. In cells without a cell wall (animal cells and protozoa), water chemistry influences cytolysis, whereby osmotic pressures cause cell lysis if not maintained. This converts cellular DNA to dissolved DNA (Figure 1) and has been discussed in refs (12 and 35). Conversely, the release of DNA from cells with cell walls (plant cells) results from enzymatic breakdown of the polysaccharides and lignin composing their structure.^36^ Thus, the activity of extracellular microbial enzymes is likely the rate-determining step in plant cell lysis. The activity itself increases with increasing enzyme concentration and is sensitive to both temperature and ultraviolet (UV) light exposure.^37^ Inside eukaryotic cells are cytoplasmic organelles that contain mitochondrial and chloroplast DNA and consist of a double lipid bilayer membrane and, like animal cells, undergo similar lysis processes.

### Adsorption–Desorption

The backbone of DNA contains negatively charged phosphodiester groups which play a key role in DNA adsorption to mineral and organic particle surfaces. At circumneutral pH, DNA is electrostatically attracted to positively charged mineral surfaces, such as those of iron (oxyhydr-)oxides and aluminum (hydr-)oxides, resulting in strong adsorption.^21,25,38−41^ Conversely, DNA is electrostatically repelled from negatively charged surfaces, including silicon dioxide or the basal planes of some clay minerals.^22,25,42,43^ Therefore, the importance of adsorbed DNA in a water sample likely increases with increasing suspended amounts of positively charged minerals. Electrostatic DNA–sorbent interactions can be modulated by solution pH for sorbents that carry a variable charge: increasing pH decreases the positive charges (and increases the negative charges), thereby weakening electrostatic attraction. Thus, increasing solution pH is expected to lower DNA adsorption and can facilitate DNA desorption from variably charged surfaces.

DNA–sorbent electrostatic interactions are also modulated by solution ionic strength and composition. Increases in solution ionic strength attenuate both DNA electrostatic attraction to and repulsion from positively and negatively charged surfaces, respectively. At very high ionic strength, electrostatic repulsion from negatively charged surfaces may be attenuated to an extent that close-contact DNA–surface attractive interactions (see below) result in DNA adsorption. The presence of divalent cations in solution may lead to increased adsorption to negatively charged sorbents via “cation bridging” between the like-charged DNA and the sorbent.^44−47^ Therefore, information on the solution ionic strength and concentrations of Ca^2+^ and Mg^2+^ is important to assess the extent of DNA adsorption. Besides electrostatic interactions, DNA–surface van der Waals interactions and H bonding may drive adsorption. However, these energetic contributions are expected to be small in comparison to electrostatic interactions.

All of the aforementioned interactions result in “physisorption”—the interaction of DNA with the sorbent surface without forming covalent bonds. However, DNA may additionally bind to some surfaces through “chemisorption”, which involves the formation of covalent bonds between the phosphodiester group of the DNA and hydroxyl groups on the mineral surfaces. The resulting “inner sphere” complexes are very stable and may both result in DNA adsorption to mineral surfaces even at high pH (despite net negative surface charges on the minerals) and prevent DNA desorption from mineral surfaces even if changes in solution conditions result in DNA–sorbent electrostatic repulsion. DNA may thus be irreversibly adsorbed, which is clearly relevant for eDNA decay and detection.

Finally, cosolutes may compete with DNA for adsorption sites on particle surfaces and thereby suppress DNA adsorption. For instance, both dissolved organic matter (DOM) and phosphate are expected to adsorb to some mineral surfaces and may thus increase the fraction of eDNA present in the dissolved state.^25^

## Expected and Observed Decay Processes of eDNA

### Expected Decay Processes

Chemical reactions of DNA may alter its size and modify its chemical structure, both of which determine its detectability in aquatic samples (see the Appendix). Chemical reactions include photochemical oxidation, abiotic hydrolysis, and enzymatically mediated hydrolysis (which we refer to as biological degradation since these enzymes are produced by living organisms). Both enzymatic and abiotic reactions cause hydrolytic cleavage of ester bonds in the backbone of DNA and result in the conversion of a longer DNA molecule into shorter molecules. Physical shearing of DNA molecules is also a potential mechanism, but these forces are unlikely in natural aquatic systems (Appendix). The importance of these reactions for using eDNA to infer the species’ presence is that eventually these short molecules can no longer be detected by the use of methods such as PCR. It is assumed that hydrolysis of eDNA can occur both intracellularly and extracellularly (Figure 1), thus affecting multiple eDNA states. Abiotic hydrolysis or photochemical oxidation is likely easier to predict (based on readily measurable chemical parameters such as solution pH and UV light irradiance) than enzymatic hydrolysis, which requires more detailed information concerning type, abundance, and activity of the enzymes as well as the population dynamics of the microorganisms secreting these enzymes. Further, microbial activities (e.g., demand for phosphorus) are expected to be sample- and time-specific and may require assessment when a water sample is collected.^10^ Adsorption of nucleic acids to particle surfaces has been shown to stabilize these molecules by protecting them from hydrolytic enzymes in water.^48−51^ Likewise, there is evidence that particle adsorbed DNA is protected from photochemical degradation.^52^ Thus, once DNA is bound to surfaces of minerals, it is expected to be stabilized from degradation.

### Observed Decay Processes

**Figure 2:**
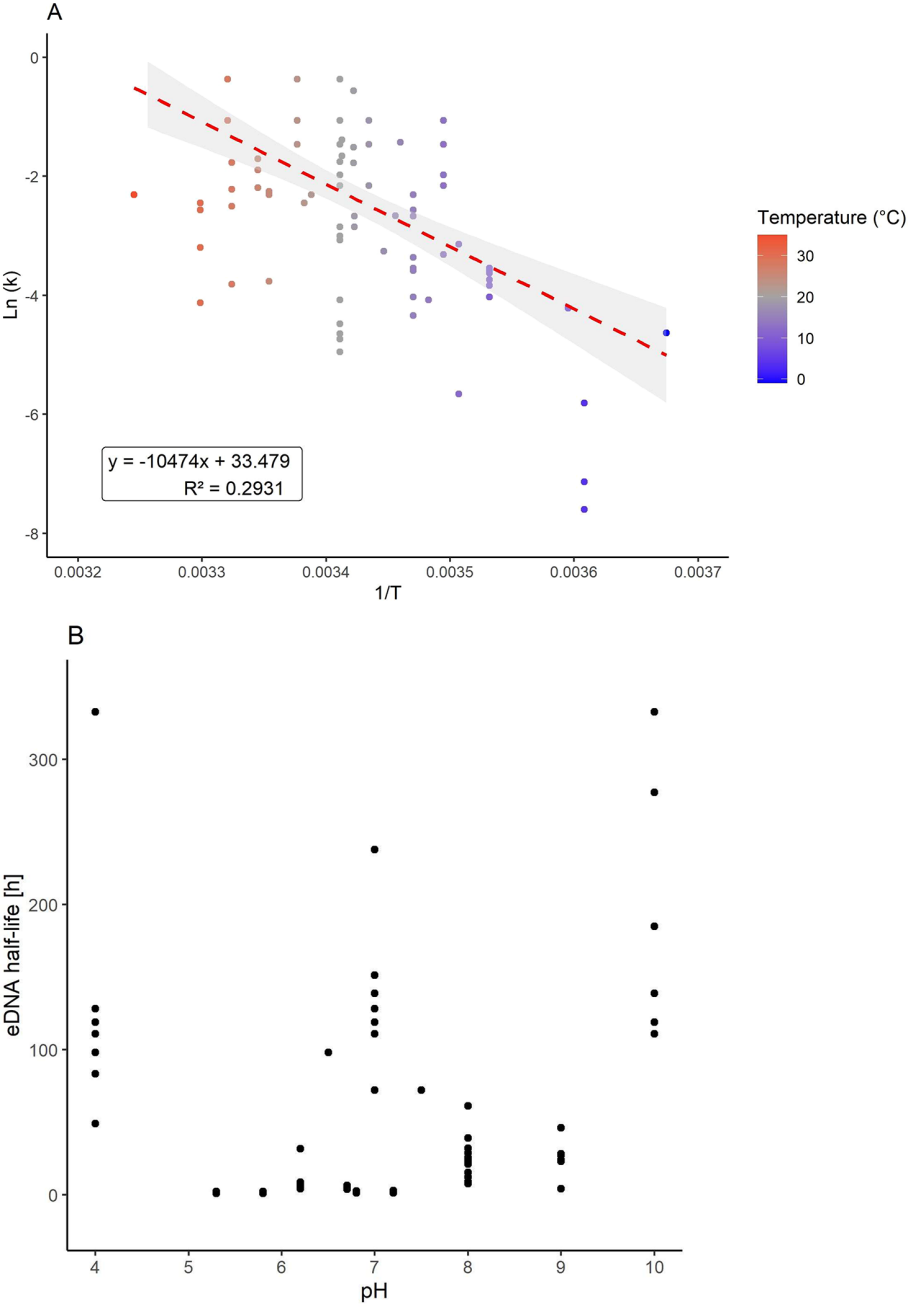
(A) Fish eDNA decay in relation to temperature. Only data for marine and freshwater fish were included. The natural logarithm of the decay constant *k* is plotted against the reciprocal values of the temperature expressed in kelvin [1/*T*], analogous to the temperature dependence of reaction rates presented in the Arrhenius equation. (B) eDNA data from amphibians, fish, and crustaceans in relation to water pH. eDNA half-life (in hours) is plotted against pH of water where the organisms were present. Data and associated articles used for (A) and (B) can be found in Tables S4 and S5, respectively. The figure was generated using the R package ggplot2 v3.3.3.

In aquatic systems, the reactions expected to lead to DNA decay are likely further influenced by the state that eDNA assumes.^11^ We synthesized data from the eDNA literature (see Tables S1–S3) and conducted a meta-analysis (see Tables S4–S6) to evaluate what is known about eDNA decay processes based on temperature, pH, and microbial activity. The meta-analysis conducted for generating Figure 2 and Figure S1 was independent from the Web of Science and literature search and synthesized data (see Tables S1–S3) detailed in the next section. Data for Figure 2 were extracted from the meta-analysis conducted in ref (57), and all extracted data were verified in the original publications. Additionally, a Google Scholar search was conducted in October 2020 searching for the terms “environmental DNA” or “eDNA” together with “degradation” or “decay” and “temperature” or “pH”, resulting in the addition of data from ref (58) to Tables S4–S6. Data from refs (59 and 60) were added at a later stage of the analysis. Values for eDNA half-life in hours were directly extracted or calculated from the reported first-order decay rate constant. Data from marine and freshwater organisms, namely fish, crustaceans, amphibians, and insects, were included in our analysis. However, only values from marine and freshwater fish are displayed in Figure 2A, while data from amphibians, fish, and crustaceans are displayed in Figure 2B. Additional taxa are displayed in Figure S1. Based on this meta-analysis, exponential decay functions are increasingly fit to experimental DNA decay data showing that, independent of source organism, eDNA decay exhibits a pattern of first-order kinetics. Yet, some studies also demonstrate that a second-order (or biphasic) decay rate constant better describes the observed eDNA data.^61,62^ As suggested by Jo et al.,^11^ the need to fit a biphasic decay rate constant to observed experimental data may indicate that different rates may be associated with different eDNA states. However, because PCR detection of DNA cannot differentiate between states, the first-order decay rate constant is likely an integrated estimate for eDNA decay across multiple states contributing to detection. The integrated estimate may be good if the question is “was this species ever present in this ecosystem?”, but integrating across states with unknown persistence times in the environment can decrease the accuracy of this inference if a finer temporal resolution of species presence is sought.

Broadly, observations are that eDNA rate constants of decay increase with increasing temperatures (>20 °C) but decrease with more basic (pH >5.0) or alkaline solutions (pH >9.0) (Figure 2, Tables S4 and S5). Furthermore, studies to date (see Tables S1 and S2) include both seminatural and experimental aquatic systems but have thus far measured animal eDNA (especially fish), leaving much to be explored for what happens to plant and other animal eDNA in the water column. Enzyme kinetics depend on the same parameters that affect abiotic DNA decay, for example, temperature, pH, UV-B light irradiation, and cofactors such as metal ions that either enhance or inhibit enzymatic activity.^63^ Thus, we would expect these environmental parameters to be highly correlated with eDNA decay rates (*k* ranging from 0.0005 to 0.693) whether or not enzymes are involved. A single study has comeasured eDNA in different states (cell versus dissolved DNA) and found differences in the decay rates between states for pond water but not salt water.^60^ This suggests that water chemistry in different habitats may play a role in the degradation of different states. Finally, it is still uncertain whether fragment size of amplified DNA could have an impact on the eDNA decay rates. While Rees et al.^1^ advocates that small fragment sizes are likely to persist longer, and Jo et al.^64^ found that long DNA fragments showed higher decay rates than short ones, Bylemans et al.^65^ found no evidence that larger eDNA fragments have a higher decay constant. Andruszkiewicz et al.^66^ therefore recommend that size fractionated studies are used in conjunction with shedding and decay experiments to elucidate their impact. Here, Figure 2A highlights the temperature dependence of the eDNA decay following the Arrhenius law equation. We acknowledge that this first-order model might oversimplify the relationship of temperature in the eDNA decay processes, and it should be noted that other studies have hypothesized that other decay models besides a log–linear one could account for transition between the different eDNA states.^61,65,66^

Lastly, microbial abundance and activity are expected to play an important role in animal and plant eDNA decay in water (ref (9) and references therein). While studies have been performed on soil and sediments,^67−70^ no systematic experiment has been conducted to determine the relative importance of abiotic versus biotic DNA degradation in water. Several studies have suggested higher microbial activity contributes to the faster DNA degradation observed at higher temperatures,^35,54,57,71^ which appears to be supported by a mesocosm experiment that examined the influence of microbial activity on fish eDNA degradation. However, the experiment did not control bacterial abundance independently of temperature or time.^72^ Another study examining bacterial abundance in relation to eDNA used radiolabeling as opposed to PCR amplification of natural seawater samples;^10^ thus results are based on total eDNA as opposed to animal and/or plant eDNA. Bacteria are known to graze on DNA for nutrients in aquatic ecosystems through extracellular enzymes and ectoenzymes (e.g., nucleases on the surfaces of their cells that hydrolyze DNA^13,53^). Active DNA-degrading enzymes have been found in filtered water fractions containing bacteria, cyanobacteria, algae, fungi, and single-cellular and multicellular plankton animals, but some enzyme types (e.g., 5′-nucleotidase) have only been found on surfaces of bacteria cells.^73^ Another study employed antibiotics to decrease bacterial loads and found that antibiotics decreased eDNA decay rates to smaller values than measured under higher bacterial loads in untreated samples,^56^ suggesting that microbial decay is the main driver. However, in both of these studies, there was no control without bacteria to determine the relative importance of abiotic reactions. If enzymes secreted by cells are the main driver of hydrolysis of DNA, the subsequent nutrient utilization (N and P) by microbial cells is a plausible mechanism for the shorter decay rates (hours to days) observed for animal eDNA in natural water compared to abiotic reactions which occur over much longer time scales (Appendix).^10^ This would lead to environment-specific rates of eDNA decay requiring an understanding of both N and P limitation and the parameters that control the eDNA state (discussed under State Conversion Processes).

## Identifying Information Needed to Understand eDNA States, Decay, and Implications for Species Detection

We have discussed four eDNA states (dissolved, particle absorbed, intracellular, interorganellar) from eukaryotic organisms that are likely to be present in aquatic environments. Processes responsible for conversion between states and eDNA decay are detailed and well understood. Studies of eDNA decay in natural and artificial aquatic systems to date provide evidence that environmental parameters affect DNA decay rates in water.^5^ We have made the case that chemical reactions that cause eDNA decay are likely to be state-specific and decay rate constants are influenced by the physical and chemical properties of aquatic environments. Thus, the next step is to form a greater understanding of what states are present for analysis in natural systems. With this in mind, a synthesis of published eDNA studies targeting single species was undertaken to investigate whether we could ascertain what eDNA states are being analyzed overall, and whether the detection of the species’ DNA from a specific environmental context could inform which eDNA state was present. A Web of Science literature review targeting species-specific eDNA studies in aquatic habitats was conducted in March 2020 (see details of the search and analysis in the Supporting Information). This focus simplified the relationship between DNA and its dynamics by looking at a single species in a system and avoided potential metabarcoding biases. We note that this literature review may potentially be biased by methodologies that resulted in a positive eDNA detection from water samples (as nondetections are less likely to be published). We concentrated on methods used to isolate eDNA from a water sample and inferred what states were likely analyzed. Because of the chemical properties of eDNA states, we know that molecular purification protocols can select and potentially isolate different states from a water sample. Additionally, we recorded environmental parameters that were comeasured at the time of sampling. From a total of 419 indexed peer reviewed articles, 59 were retained, and seven more articles published in *Environmental DNA* (nonindexed at the time of the search) were added, resulting in a total of 66 articles. Following this, 76 predefined variables were recorded following a standardization procedure (see the Supporting Information) and synthesized using bar plots or Sankey diagrams, except variables where values ranged widely. Taxonomic groups targeted by assays, applications of assays, environments where assays were used, and geographic deployment of assays are summarized in Figure S3.

**Figure 3:**
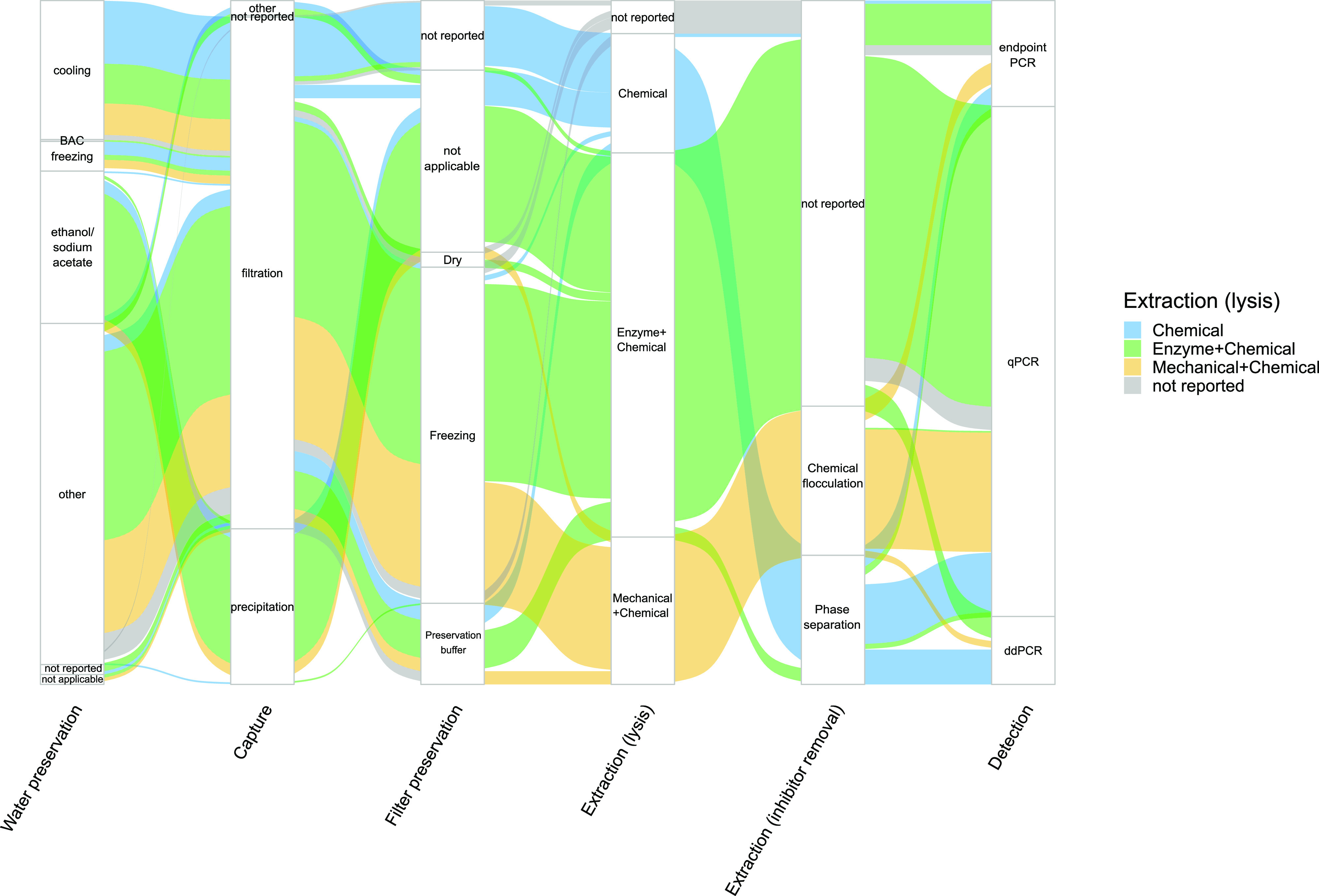
Sankey diagram summarizing the methodological flows of assays through water preservation, capture, filter preservation, extraction (lysis and inhibitor removal), and detection in synthesized studies. Direction of flows are from left to right. Sizes of flows are proportional to the number of assays using a particular method. Flows are colored by the method used for lysis during DNA extraction as this is most likely to influence the state of eDNA being analyzed downstream. Note that BAC means benzalkonium chloride, qPCR means quantitative polymerase chain reaction, and ddPCR means droplet digital PCR.

We found that most eDNA studies are broadly employing the same molecular methods for DNA capture (i.e., filtration), extraction (i.e., enzyme and chemical), and detection (i.e., qPCR), albeit in different combinations (Figure 3). Most assays used cooling (*n* = 25) after “other” (*n* = 58, typically centrifugation or resin beads) for water sample preservation, followed by filtration (*n* = 126; Figure 3, Figure S4) for eDNA capture. Ethanol/sodium acetate (*n* = 5) for water sample preservation followed by precipitation (*n* = 31) for eDNA capture was less popular but constituted a second major methodological workflow (Figure 3, Figure S4). Of those assays using filtration, glass fiber filter membranes (0.7 μm pore size) were most commonly used, followed by polycarbonate track-etched, cellulose nitrate, nylon, and “other” membrane types, including cellulose acetate and poly(ether sulfone) (Figure S5). Filters were typically frozen at −20 °C for preservation of DNA in the retentate (Figure 3), but storage times were often not reported. A full breakdown of precipitation and filtration methods can be found in Figures S6 and S7.

The vast majority of assays (*n* = 125) used commercial extraction kits (82.0%) as opposed to unbranded protocols (18.0%) (Figure S8), with the Qiagen DNeasy Blood and Tissue Kit being the most commonly used (47.76%; *n* = 110) (Figure S9). Mechanical disruption with chemicals and chemicals only were secondary to an enzymatic digestion with chemicals for cell lysis (Figure 3). Commercial kits typically employed an enzyme with temperature to induce cell lysis and lacked an inhibitor removal step, yet postextraction inhibitor removal was uncommon (Figure S10). Where postextraction inhibitor removal was performed, this was done by either phase separation or chemical flocculation (Figure 3) using methods such as the Zymo One Step PCR Inhibitor Removal Kit, the Promega Wizard Genomic DNA Purification Kit, chloroform, and dilution.

Most assays (*n* = 136) targeted mitochondrial genes by using quantitative PCR (69.9%) (Figure 3, Figure S11). Technical replication, reaction volumes, volume of template DNA, inclusion of an internal positive control to test for inhibition, determination of the limit of detection and the limit of quantification, and assessment of environmental matrix effects for assays are summarized in Figure S12. Master mixes and enhancers used with assays are summarized in Figures S13–S15. Crucially, most assays (*n* = 145) did not measure, record, or report environmental parameters that are expected to affect the distribution of DNA among states and determine the stability of DNA (Figure S16). Parameters that were recorded and reported included temperature (50.3%), pH (22.0%), UV exposure (9.0%), season (68.0%), canopy cover (3.0%), conductivity/salinity (22.0%), geology of catchment (12.0%), and dissolved oxygen (15.0%). One-third of papers would require the authors to be contacted to clarify their analytical workflow or ascertain if they collected environmental data but did not report it (Figure S17).

Taken all together, our synthesis suggests that most single-species studies employ methods that analyze a similar and potentially restricted state of eDNA. The majority of studies use filtration at pore sizes through which most dissolved eDNA may pass if clogging does not occur. After filtration, the eDNA on the filter is isolated with similar lysis methods and purification buffers provided with commercial extraction kits that fundamentally employ similar chemistry (see Table S2). Most of these commercial kits likely do not promote particle bound DNA to desorb. To be certain, the constituents of the buffers would need to be determined, which was not feasible since most of these are trade secrets. If these commercial kit buffers do not have competitive binders and do not reach a pH high enough to promote desorption, it is likely that DNA adsorbed onto particles was not isolated. If these assumptions are true (i.e., dissolved DNA flows through filters and extraction kit buffers do not promote desorption), then eDNA detections from the studies reviewed here may originate from only intercellular or organellar DNA. However, extensive research comparing whether specific molecular methods copurify multiple eDNA states would be needed to verify this claim.

## How Do We Create Analytical Controls for State?

The importance of appropriate analytical controls in eDNA research is well-established.^15,74^ These include field and laboratory controls that are designed to assess contamination (negative controls),^75^ analytical precision (biological and technical replicates), and sensitivity (positive controls). However, these controls do not account for eDNA being present in different states, nor do these controls allow assessment of whether eDNA in each state (or states) is accurately quantified. Moreover, incomplete recovery of analytical controls typically leads to the conclusion that PCR inhibition is involved. While this clearly is a possibility, we propose that results could also be confounded because current protocols may not completely extract DNA from all four states if present in the sample. Therefore, additional analytical controls are needed to disambiguate the cause of observed signal attenuation (e.g., PCR inhibition versus inefficient extraction across states). These controls and when to add them to the workflow do not yet exist. We provide some ideas next, but more research into how to do this needs to be undertaken.

**Figure 4:**
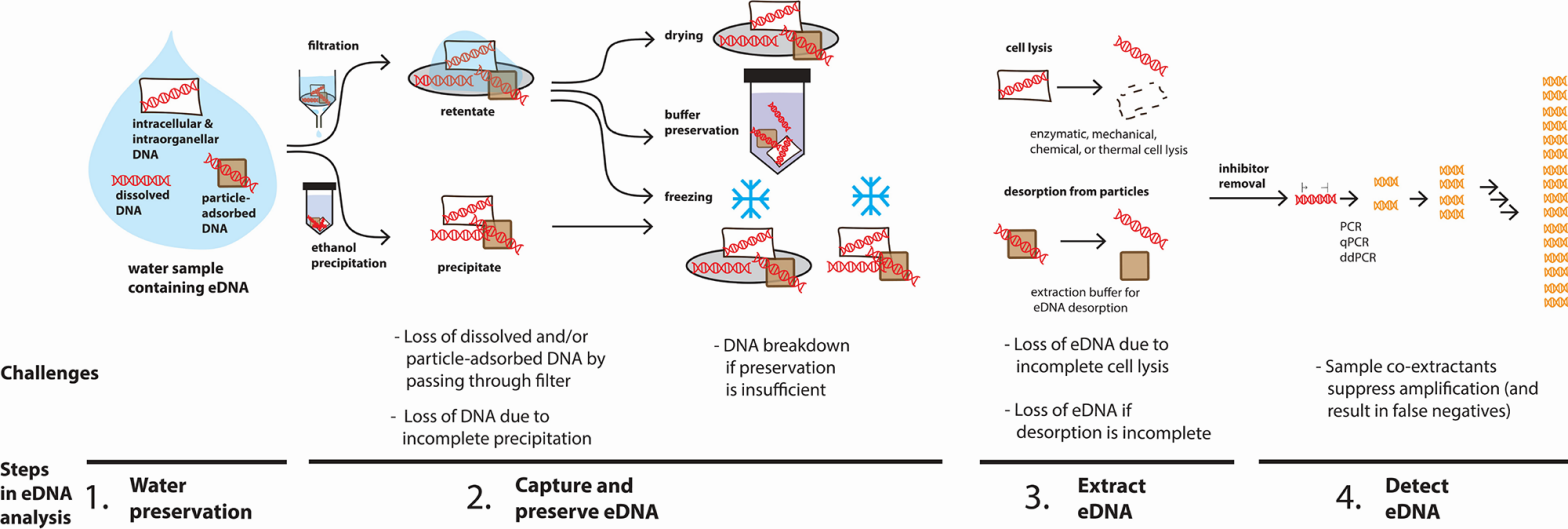
Sample processing steps used for water preservation, eDNA capture and preservation, eDNA extraction, and eDNA detection. Each step can be done in several ways, and each can likely have a challenge associated with loss of a particular eDNA state.

There are various analytical controls employed in the eDNA literature, but these are inconsistently applied. Some researchers (e.g., ref (76)) advocate multiplexing an assay for a given target species together with an assay designed to detect a co-occurring species presumed to be ubiquitous in the environment, such as algae (e.g., using a generalized plant chloroplast DNA assay), to demonstrate that the PCR reaction was not inhibited. Yet, because the state (Figure 1) and concentration of any species’ eDNA is unknown, this general marker cannot be used to assess relative rates of PCR inhibition or whether nondetection is the result of inefficient eDNA recovery. To address this issue, internal standards of known DNA concentration and state could be applied at various stages in the workflow (Figure 4). Synthetic DNA has been used as an internal positive control to quantitate the relative degree of PCR inhibition, but this does not account for inefficient extraction of different eDNA states. Applying a “spike” in control prior to the extraction/precipitation step could result in some sorption of the control DNA, but again the attenuation of the PCR signal could not be used to discriminate between inhibition and inefficient recovery.

Developing analytical controls to assess whether eDNA is bound to cellular debris, adsorbed to particles, or dissolved in solution remains a challenge. Size fractionation can be achieved by filtering a sample through multiple filters of progressively smaller pore size and subsequently extracting eDNA from each individual filter and the filtrate. Assuming any DNA that passed through the filters into the filtrate represents dissolved eDNA and potentially even particles, it is possible to quantify this pool. However, eDNA recovered from the filters cannot be separated into cellular bound versus particle bound DNA without utilizing extraction protocols optimized to recover only particle bound or cellular debris bound eDNA. Protocols for separating soluble DNA (i.e., extracellular and bound to particles) from insoluble DNA (i.e., still inside the cell) have been developed.^77^ Their parallel application to known mixtures of cellular bound, particle bound, and dissolved eDNA could prove illuminating by separating out the different states and analyzing them separately for detection of a target species or community. However, quantifying the eDNA in each category before assembling the mixtures would be nontrivial, and even then, the approach could not easily assess the dynamic conversion of DNA between states that may occur during the extraction. These issues notwithstanding, the combined use of cellular material, plasmids (e.g., as surrogates for organelles), synthetic DNA, and varied adsorbent materials, together with size fractionation and multiple extraction techniques as applied across a gradient of environmental conditions, could yield novel insights concerning extraction efficiency among eDNA states and the dynamic conversion processes between them when selectively applied to each sample processing step (Figure 4).

## Recommendations for Analytical Procedures and Future Experiments

A growing body of literature demonstrates that eDNA-based detection is a powerful, sensitive, and noninvasive method of biodiversity detection, yet the extent to which existing methods may be susceptible to inefficiencies remains to be systematically investigated. It is evident that at least four states of eDNA exist and not all of them may be captured by the various combinations of methods (Figure 3) used to isolate DNA from water.

To maximize detection rates, methods that capture and isolate DNA from all states should ideally be utilized, such as water filtration using different pore sizes (i.e., to capture particle bound or cellular DNA and avoid clogging the filter) followed by precipitation of the filtrate (i.e., to capture dissolved DNA). Specifically, adsorption effects should be considered when capturing and extracting eDNA from turbid waters, or in the presence of highly concentrated suspended solids as on a filter. A side effect of DNA extraction is the release of intracellular DNA during cell lysis which could encounter positively charged mineral surfaces that were cocaptured during filtration, resulting in the newly released DNA becoming particle bound during extraction and subsequently in reduced DNA yield. In such cases, extraction buffers that effectively extract DNA from mineral surfaces will need to have the corresponding compositions to favor desorption and prevent adsorption of DNA liberated from cells. In particular, these extraction buffers should (i) have a sufficiently high (i.e., alkaline, pH 9–10) pH to result in DNA–sorbent electrostatic repulsion, but not too high to facilitate base-catalyzed DNA backbone hydrolysis; (ii) contain competing coadsorbates such as phosphate, pentaphosphate, or possibly a DNA molecule that does not contain the targeted sequence of the analyte DNA; and (iii) contain complexing agents for divalent cations to minimize the possibility of cation bridging of DNA to negatively charged sorbent surfaces. Notably, extraction protocols developed for soil and sediment may be more efficient for the extraction of eDNA from water with a high concentration of particles whose surfaces can adsorb DNA, particularly if these particles are concentrated with eDNA during filtration.^78^ A systematic DNA extraction assessment using artificial control samples with known concentrations of freely dissolved, particle-adsorbed, and intracellular DNA is needed to determine which states are most efficiently captured by common extraction protocols. This would aid optimization of the extraction protocol and account for the different eDNA states while maximizing their extraction efficiency.^5,79^

Where possible, we recommend that eDNA practitioners employ methods to capture multiple states of eDNA. All samples should then be combined for analysis or analyzed independently if eDNA states are likely to influence the research or management questions under investigation, (e.g., inferences of where and when a species was present^3^). If it is impossible to extract all eDNA states at every study site, we highly recommend that eDNA practitioners resample sites that are suspected false negatives should their chosen methods of eDNA capture and extraction (most likely filtration and a commercial DNA extraction kit) fail to produce eDNA detections. Doing so will minimize false negative detections and reduce the negative impacts of such results on conservation management plans or infrastructure. However, a caveat to the above is that if different states have different decay rates (e.g., particle bound DNA might persist longer than dissolved DNA), then the time and space inference as to when a species was present in the sampled environment becomes less clear. Thus, for accurate inferences of time and space, not just detection, more research is required to determine concentration dynamics for all eDNA states present in different ecosystems.

## Conclusions

Environmental DNA exists in a mixture of different states (e.g., dissolved, particle adsorbed, intracellular, and organellar), and each state is expected to have a specific decay rate that depends on the complex interplay of varied environmental parameters. Our effort to provide a comprehensive review of the parameters affecting state-dependent eDNA decay rates and the mechanisms involved have yielded some important insights. Notably, water chemistry and suspended mineral particles likely affect conversion of eDNA among states and persistence of eDNA states in the water column. However, the eDNA literature contains inconsistently reported metadata and sometimes conflicting results; thus further study of how environmental parameters affect eDNA state conversion and decay in aquatic environments is needed. Improving our understanding of these issues will require a concerted effort by the scientific community to collect more comprehensive and consistent metadata on environmental conditions at the time of sampling. It will also require the implementation of analytic eDNA controls during sample collection, preservation, extraction, and analysis to better understand eDNA state conversion and decay in aquatic environments. We make the case that these controls are not yet developed, and until this is the case, attempting to collect eDNA from many states seems warranted to reduce false negative detections when stakes are high (e.g., detection of harmful invasive species). The study of states, their persistence, and analysis represents a crucial research agenda to increase the reliability and application of eDNA detection methods. This is especially needed given the shift toward using eDNA detection methods as a tool to support management decisions pertaining to invasive alien species, species at risk, and other valued ecosystem component species.

## Appendix. Short Overview of DNA Decay by Chemical Reactions

For more details, see extensive reviews by refs (5, 16, and 53).

### Hydrolysis Reactions

DNA decay by hydrolysis can occur abiotically and be enzymatically mediated and is affected by environmental factors (e.g., water pH, temperature, and ionic strength). DNA strands break through enzymatic hydrolysis by so-called DNases. Such DNA enzymatic hydrolysis can occur at high rates and become the main driver of DNA decay as opposed to purely abiotic hydrolysis.^5,53^ Determining how environmental parameters change enzymatic hydrolysis rates is complicated by the fact that each species’ enzymes potentially exhibit optimal kinetics for different possible combinations of environmental parameters because of a species’ evolutionary history of adaptation.^54^

Abiotic hydrolysis reactions, such as depurination (loss of purine base) and deamination of cytosine (elimination of ammonia), followed by strand break cause DNA decay. Chemical depurination rates decrease with decreasing temperature, pH, and ionic strength. Deamination reactions are very slow at temperatures present for most of earth’s surface waters (excluding hydrothermal vents) and are therefore unlikely to be an important driver of DNA decay on short time scales of days to weeks.^55^ In fact, most estimates of abiotically driven hydrolysis (depurination and deamination) of DNA have half-lives between 70 and 31 000 000 years, but these can be modulated by extreme environmental conditions.^5^

### Oxidation Reactions

Radicals generated from UV-A/B light can lead to breaks in single-stranded DNA by forming hydroxyguanine and hydantoins from pyrimidines. This reaction is pH sensitive. UV light may also cause the formation of pyrimidine dimers in DNA. In addition, UV radiation has many indirect effects through the generation of free radicals; therefore the time scale upon which this mechanism acts to degrade DNA is hard to conclude.

### Physical Shearing

Alternative cycles of freezing/thawing have previously been shown to lead to progressive DNA degradation in controlled conditions^54^ (due to the formation of solid ice crystals). Additionally, DNA can be degraded through acoustic sonication or by hydrodynamic shearing in laboratory conditions, e.g., library preparation for sequencing.^56^ However, the latter two processes are least likely to occur in natural environments.

### Chemical Modification

During interstrand cross-linking, two strands of the DNA molecule become covalently linked, preventing full separation of DNA strands using heat. This can be facilitated by UV-A light and the presence of intercalating agents. Interstrand cross-linking makes DNA inaccessible to PCR detection, but this is not degradation *per se*. We mention this reaction because at this time we cannot differentiate nondetection resulting from true degradation versus interstrand cross-linking using PCR.

## Supporting Information Available

List of 66 peer reviewed articles, including assigned reviewer, authors, titles, and DOIs (XLSX)
Data retrieved from the 66 peer reviewed articles used for the literature review (XLSX)
Summary of extraction protocols used in the 66 single species eDNA studies reviewed for the present study, including a breakdown of methods and components used for the main extraction steps (lysis, inhibitor removal, binding, and washing) (XLSX)
Data and articles used for generating Figure 2A showing fish eDNA decay in relation to temperature (XLSX)
Data and articles used for generating Figure 2B showing amphibians, fish, and crustaceans eDNA decay in relation to pH (XLSX)
Data and articles used for generating Figure S1 showing eDNA decay from fish, amphibians, bacteria, crustaceans, and jellyfish in relation to temperature (XLSX)
Description of the literature review methods and analysis, and figures summarizing data from the articles reviewed for this paper (PDF)
