# Supplementary material for: The Multiple States of Environmental DNA and What Is Known about Their Persistence in Aquatic Environments
Source: Environ Sci Technol. 2022 Apr 18;56(9):5322–33. doi: 10.1021/acs.est.1c07638 (PMC9069692; doi:10.1021/acs.est.1c07638)
Supplement: Supplementary file 7 — es1c07638_si_007.pdf [file es1c07638_si_007.pdf]

Literature review methods and analysis

We conducted a Web of Science (WoS) literature search on 10th March 2020, including the terms "environmental DNA" or "eDNA" and excluding the terms "biofilm" or "biofilms" or "microbial" or "bacterial" or "microorganism" or "microorganisms" or "metabarcoding" or "metagenomics" or "next generation sequencing", restricted to articles published between January 2008 to December 2019. This resulted in 419 entries which were manually screened to ensure their suitability. We chose to focus on species-specific eDNA detection in aquatic habitats to limit the detection methodology (e.g., mainly qPCR) and simplify the relationship between DNA and its dynamics by looking at a single species in a system. We removed studies focusing on terrestrial habitats, reviews, or opinion papers, resulting in 320 articles. These articles were compared to the 327 studies analyzed in Thalinger et al. (2021), to allow for a reduction of studies which were likely to have environmental variables recorded. After this reduction, a total of 59 articles were retained as they provided details on variables potentially influencing eDNA states, methodological information relating to the state of eDNA being investigated, or variables related to the sampled systems. Furthermore, we added seven articles published in *Environmental DNA* as of December 2019 because this journal was not indexed in WoS at the time of the literature search. This resulted in a total of 66 peer-reviewed articles from which values for 76 pre-defined variables were recorded by QM, LRH, and KD. Before data collection and entry, the authors validated the same three papers to ensure standardized recording as in Thalinger et al. (2021). Data entry consisted of strings or values where variables were reported, “NR” where variables were not reported, and “NA” where a given variable was not applicable to a particular assay (e.g. probe concentration when endpoint PCR was used). Several rows of data entry were used for studies employing several methods for each aspect of the eDNA workflow (e.g. precipitation and filtration, endpoint PCR and qPCR). Finally, we retrieved information for and compared the 20 different DNA extraction methods used in these 66 peer-reviewed articles.

We summarised the data using R v3.6.3 (R Core Team, 2020). For the purposes of reporting and visualisation, a core block of variables (i.e. study ID, species, taxonomic group, amplification method, study aims) were used to distinguish an assay when examining different aspects of the eDNA workflow (e.g. filtration, DNA extraction). This was to avoid artificially inflating the number of assays employing particular methods given that information for most variables was repeated across rows where one variable differed (e.g. DNA extraction, amplification method and environmental variables were the same but multiple filtration methods were used). Most data were represented as bar plots or Sankey diagrams, except variables where values ranged widely (e.g. sample size). For these variables, the minimum, maximum

and median values are reported. Sankey diagrams were edited using Inkscape v1.0.2 (https://inkscape.org/). The R script and associated will be uploaded to a dedicated GitHub repository. The list of 66 peer reviewed articles can be found in Table S1, and the data retrieved for the meta-analysis can be found in Table S2.

Most assays (n = 100) targeted fish (49.0%), followed by crustaceans (17.0%) (Figure S3a). The majority of assays (n = 123) were used for quantification (39.8%) or detection (35.8%) (Figure S3b), and used in the Palearctic (45.3%) or Nearctic (40.7%) (Figure S3d). Assays were usually deployed in natural environments (n = 84), specifically lentic (20.7%) or lotic (26.5%) freshwater systems (Figure. S3c). Where assays were used in experimental systems (classed as 'other' for environment), the volume of these artificial water systems ranged from 900 ml to 336,000 L (median = 20 L). Sample sizes (i.e., number of sampling sites) ranged from 1 to 197 (median = 4), with between 1 and 120 biological replicates (median = 5) taken, and between 15 ml and 6 L of water (median = 500 ml) collected per biological replicate.

Typically, three technical replicates were performed in 20 µl reactions using 2 µl of template DNA. The majority of assays (80.0%) did not use an internal positive control to test for inhibition and did not determine the Limit of Detection (54.4%), Limit of Quantification (71.0%) or effects of the environmental matrix (78.0%) (Figure S12). Most assays used commercial master mixes (Figure S13), such as Applied Biosystems TaqMan Environmental Master Mix 2.0 and TaqMan Gene Expression Master Mix (Figure S14), as opposed to custom master mixes. Where custom master mixes were used, MgCl2 concentration ranged from 1.5 to 2.5 (median = 2) and dNTP concentration ranged from 0.05 to 0.25 (median = 0.20). Promega 5x Colorless GoTaq Flexi Reaction Buffer and Promega GoTaq Flexi DNA Polymerase were the most commonly used buffer type and enzyme type respectively. Enhancers were not often added to PCR reactions, but Bovine Serum Albumin (BSA) was most common where enhancers were used (21.3%) (Figure S15).

References

Thalinger, B., Deiner, K., Harper, L. R., Rees, H. C., Blackman, R. C., Sint, D., … Bruce, K. (2021). A validation scale to determine the readiness of environmental DNA assays for routine species monitoring. Environmental DNA, 3(4), 823–836. doi: 10.1002/edn3.189

Table S1. List of 66 peer reviewed articles, including assigned reviewer, authors, titles and doi numbers.

Table S2. Data retrieved from the 66 peer reviewed articles used for the meta-analysis.

Table S3. Summary of extraction protocols used in the 66 single species eDNA studies reviewed for the present study, including a breakdown of methods and components used for the main extraction steps (lysis, inhibitor removal, binding and washing). Provided as an excel spreadsheet (*TableS3_DNA_extraction_methods.xlsx*).

Table S4. Data and articles used for generating Figure 2a showing fish eDNA decay in relation to temperature.

Table S5. Data and articles used for generating Figure 2b showing amphibians, fish and crustaceans eDNA decay in relation to pH.

Table S6. Data and articles used for generating Figure S1 showing eDNA decay from fish, amphibians, bacteria, crustaceans and jellyfish in relation to temperature.

Supplementary Figures

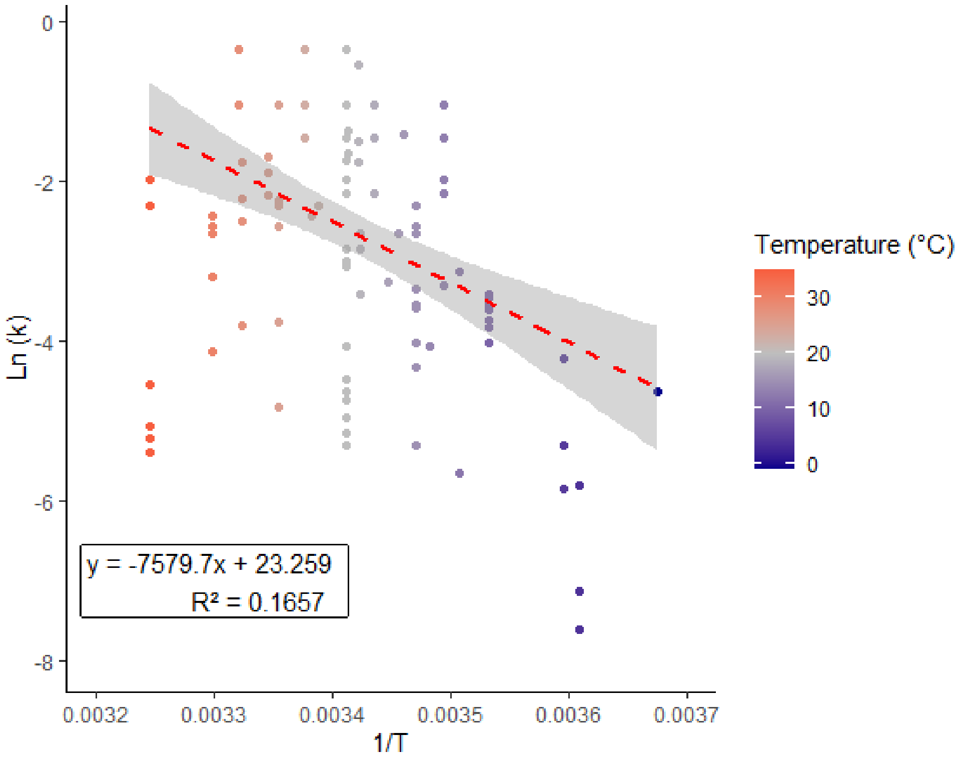

Figure S1. eDNA decay from fish, amphibians, bacteria, crustaceans and jellyfish in relation to temperature. Data from both marine and freshwater environments were included. The natural logarithm of the decay constant k is plotted against the reciprocal values of the temperature is expressed in Kelvin [1/K], analogous to the temperature dependence of reaction rates presented in the Arrhenius equation. Data were obtained from published studies where pH was kept between 6-8. When pH values were not recorded, pH was assumed to be neutral (i.e.7). Data and articles used for generating Figure S1 can be found in Table S6.

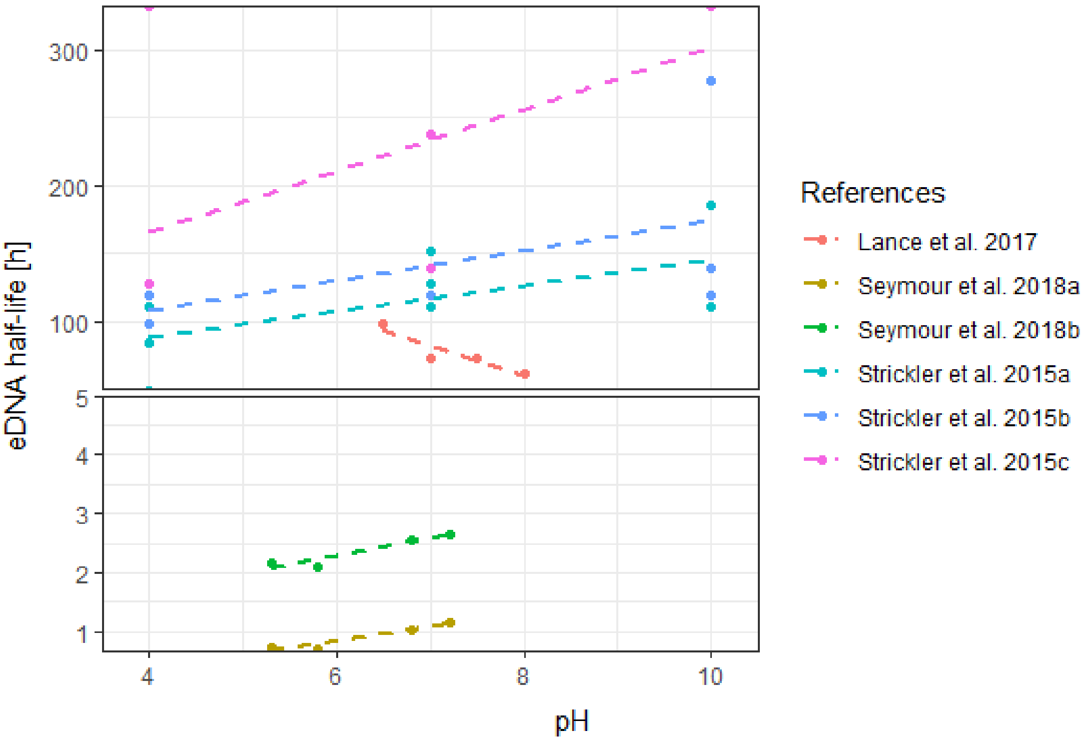

Figure S2. eDNA half-life (in hours) related to water pH using data retrieved from Lance et al. (2017), Seymour et al. (2018) and Strickler et al. (2015). Letters "a", "b" and "c" associated with the references indicate a different organism targeted within the same article.

References

Lance, R., Klymus, K., Richter, C., Guan, X., Farrington, H., Carr, M., … Baerwaldt, K. (2017). Experimental observations on the decay of environmental DNA from bighead and silver carps. *Management of Biological Invasions*, **8**, 343–359. https://doi.org/10.3391/mbi.2017.8.3.08

Seymour, M., Durance, I., Cosby, B. J., Ransom-Jones, E., Deiner, K., Ormerod, S. J., … Creer, S. (2018). Acidity promotes degradation of multi-species environmental DNA in lotic mesocosms. *Communications Biology*, **1**, 4. https://doi.org/10.1038/s42003-017-0005-3

Strickler, K. M., Fremier, A. K., & Goldberg, C. S. (2015). Quantifying effects of UV-B, temperature, and pH on eDNA degradation in aquatic microcosms. *Biological Conservation*, **183**, 85–92. https://doi.org/10.1016/j.biocon.2014.11.038

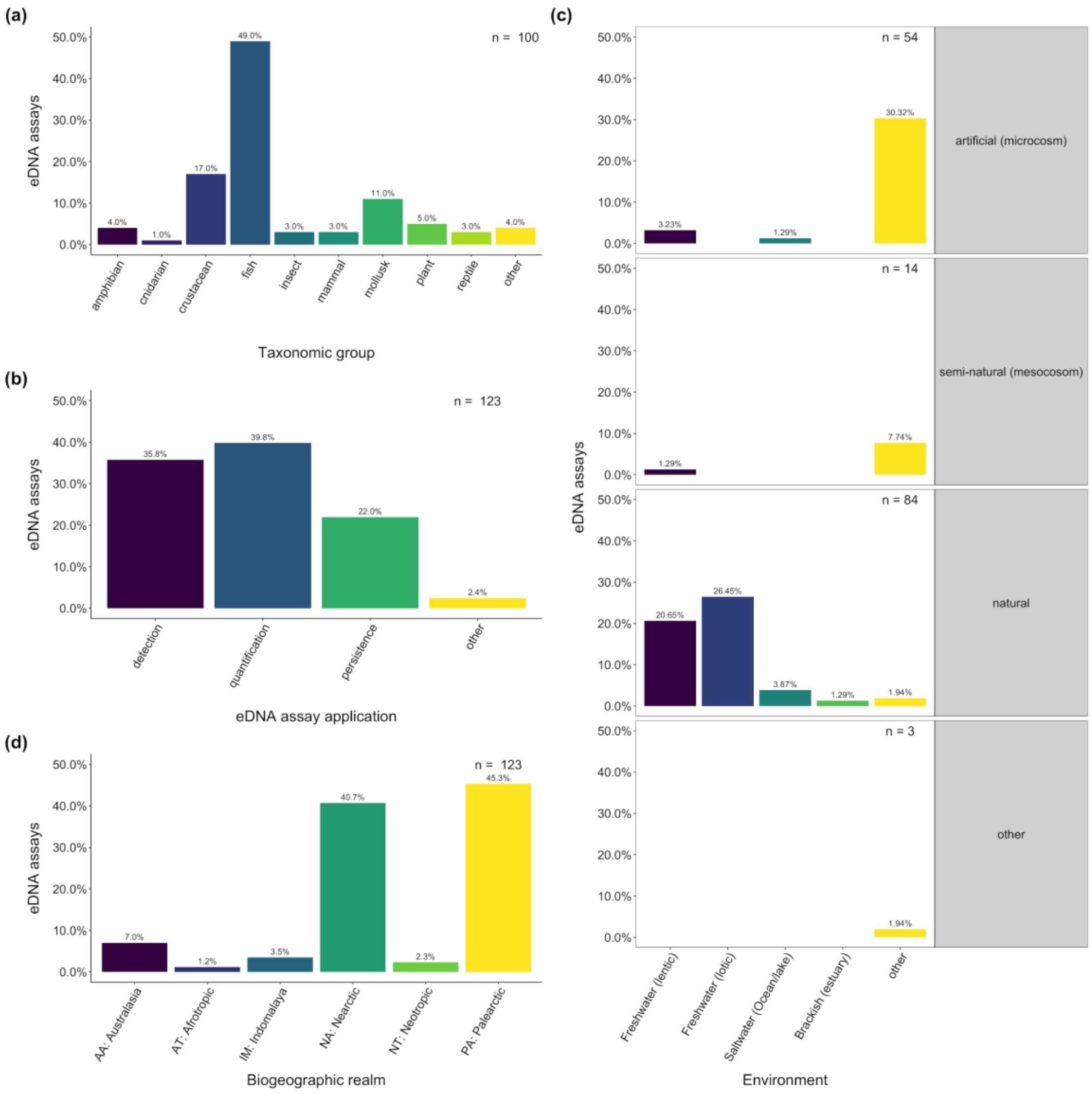

Figure S3. Bar plots summarising the number of assays that were **(a)** used to target a particular taxonomic group, **(b)** employed for a specific application, **(c)** employed in a given environment, and **(d)** used in one of six biogeographic realms. Species classed as 'other' were typically trematodes or parasites. Studies with 'other' aims were typically attempting to examine persistence and degradation in experimental systems. 'Other' environments and conditions consisted of experimental systems.

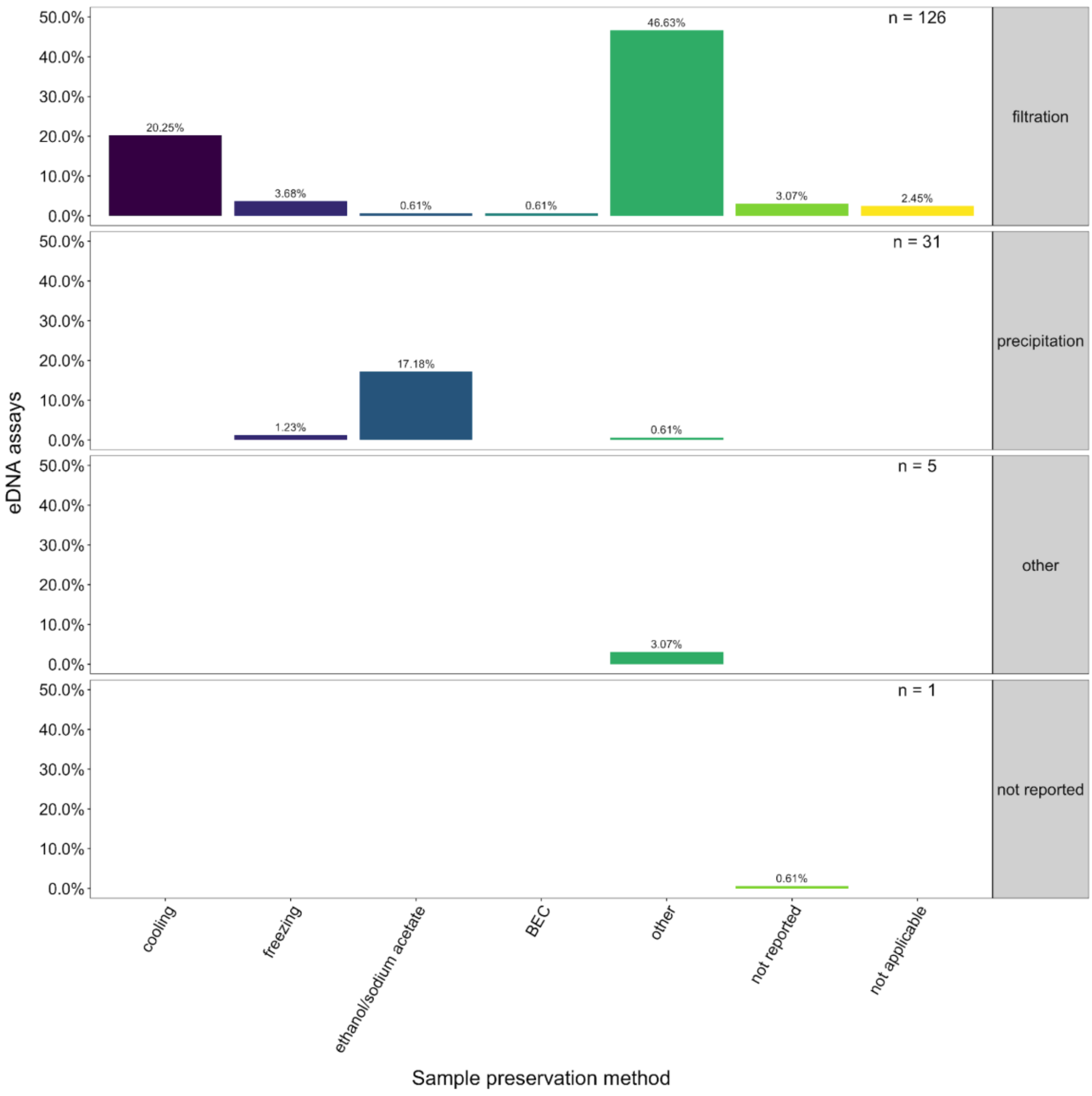

Figure S4. Bar plot summarising the number of assays that used a particular water sample preservation and capture strategy. Examples of 'other' water sample preservation strategies included on-site filtration, immediate filtration in the laboratory, and Longmire's buffer.

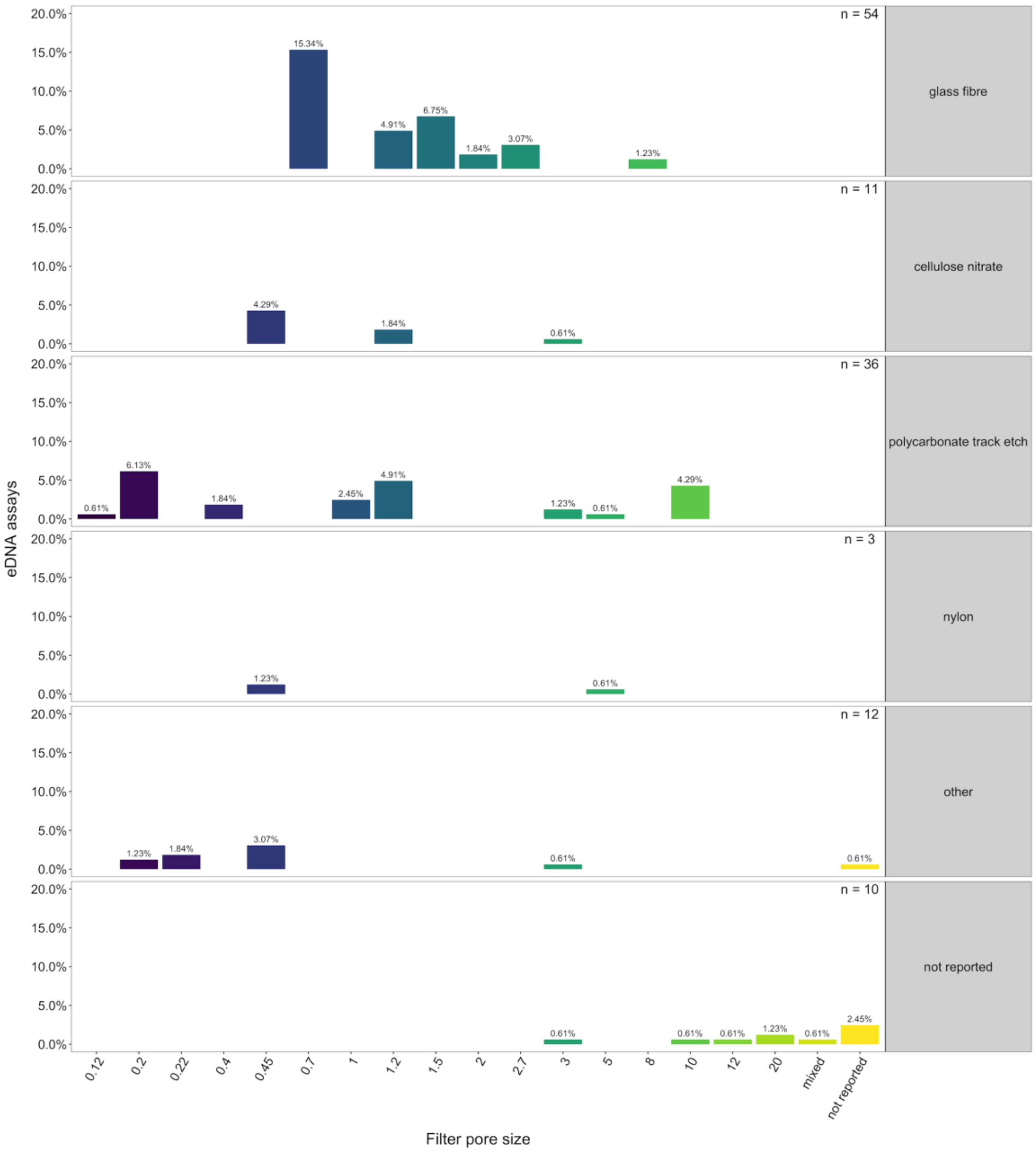

Figure S5. Bar plot summarising the number of assays that used a specific filter pore size and membrane material. Examples of 'other' filter materials include cellulose acetate and polyethersulfone.

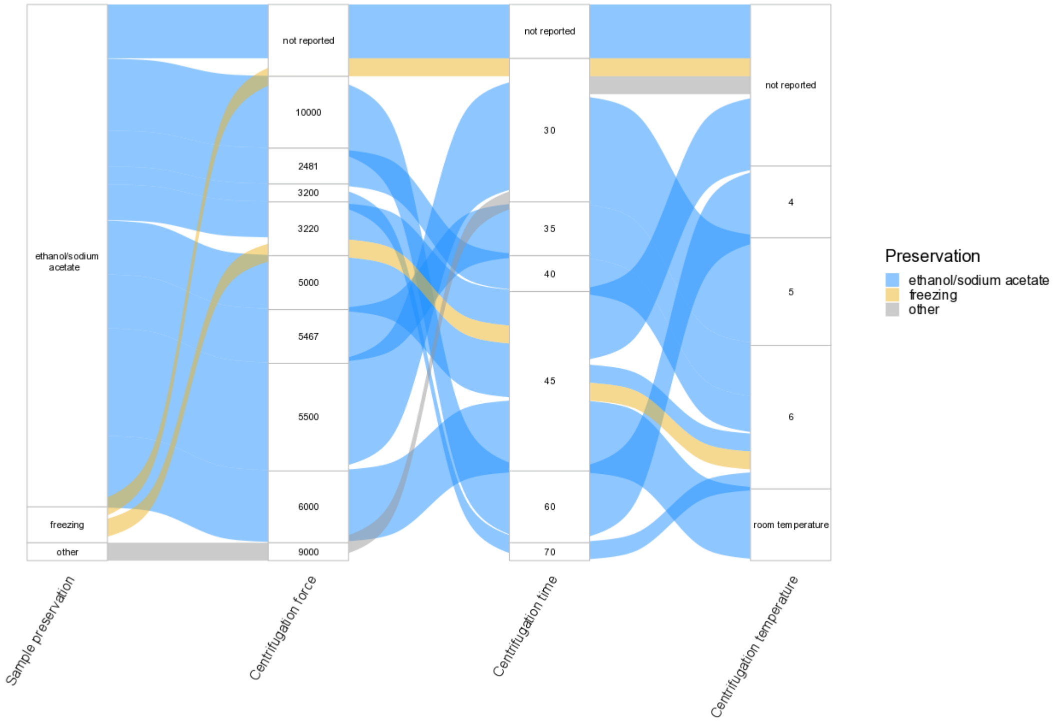

Figure S6. Sankey diagram summarising the precipitation workflows used by different assays.

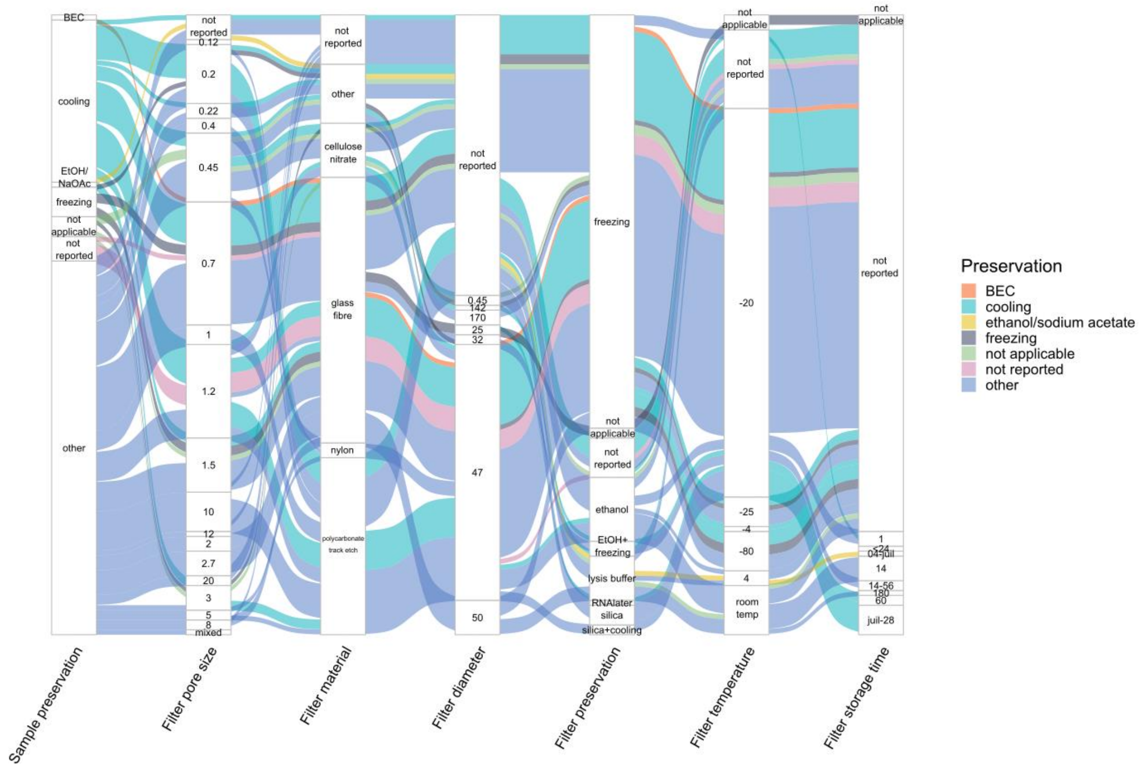

Figure S7. Sankey diagram summarising the filtration workflows used by different assays.

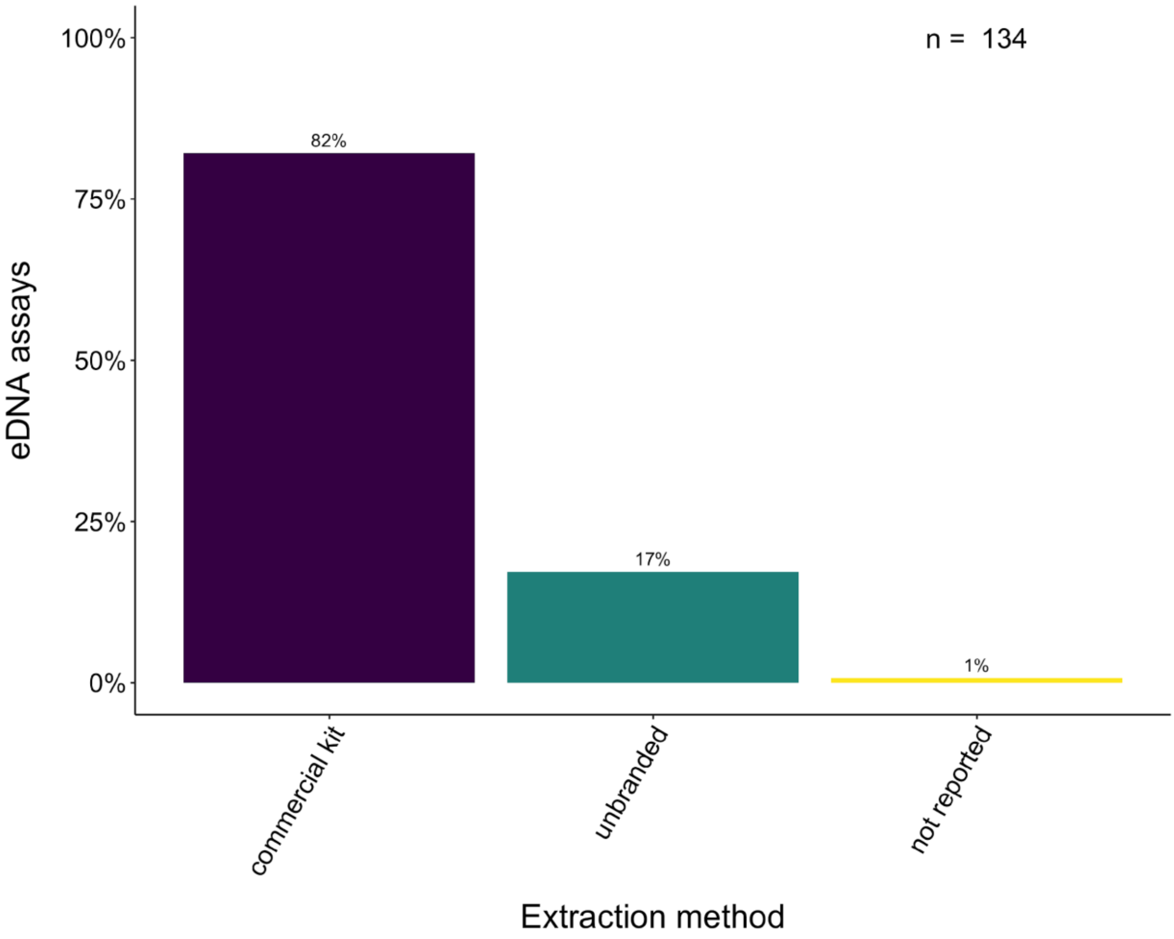

Figure S8. Bar plot summarising the number of assays that used a commercial extraction kit or unbranded protocol.

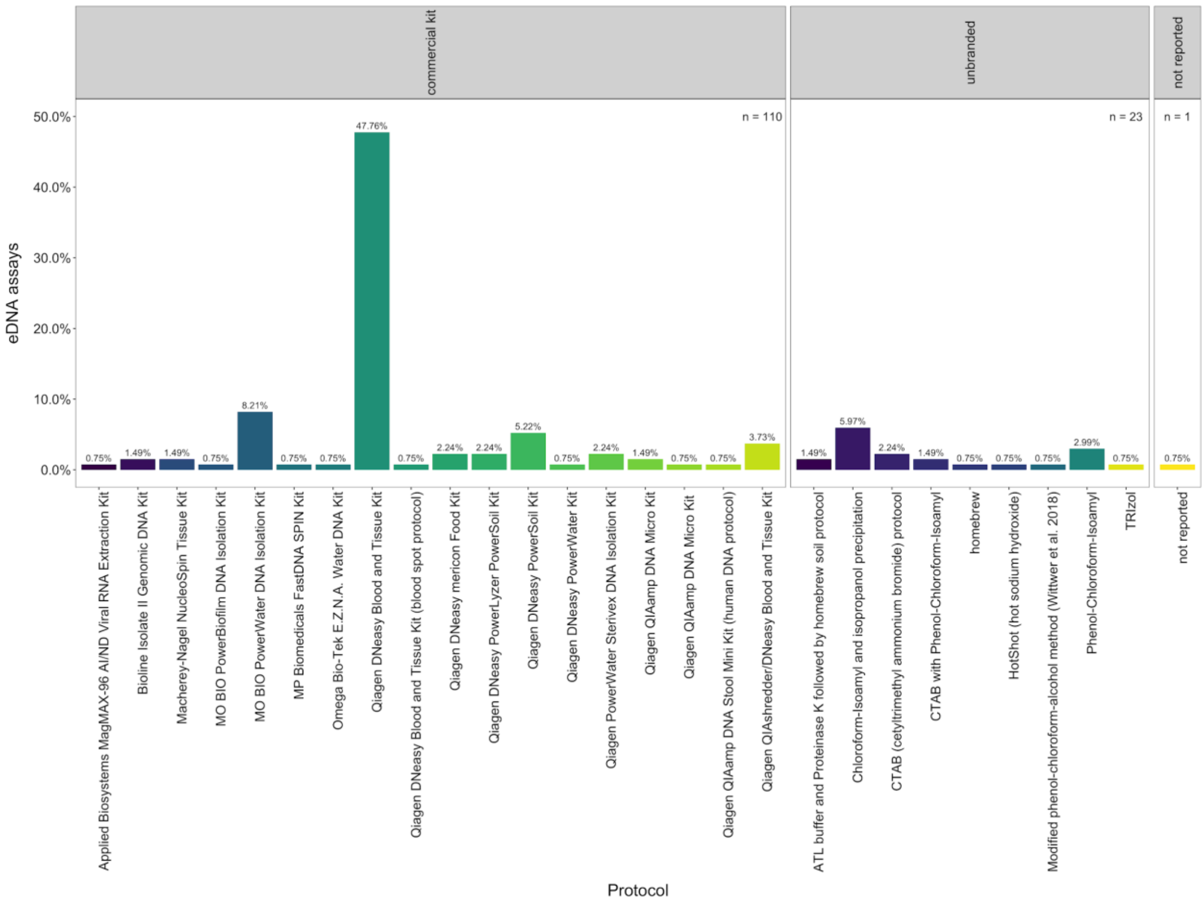

Figure S9. Bar plot summarising the number of assays that used a specific commercial extraction kit or unbranded protocol.

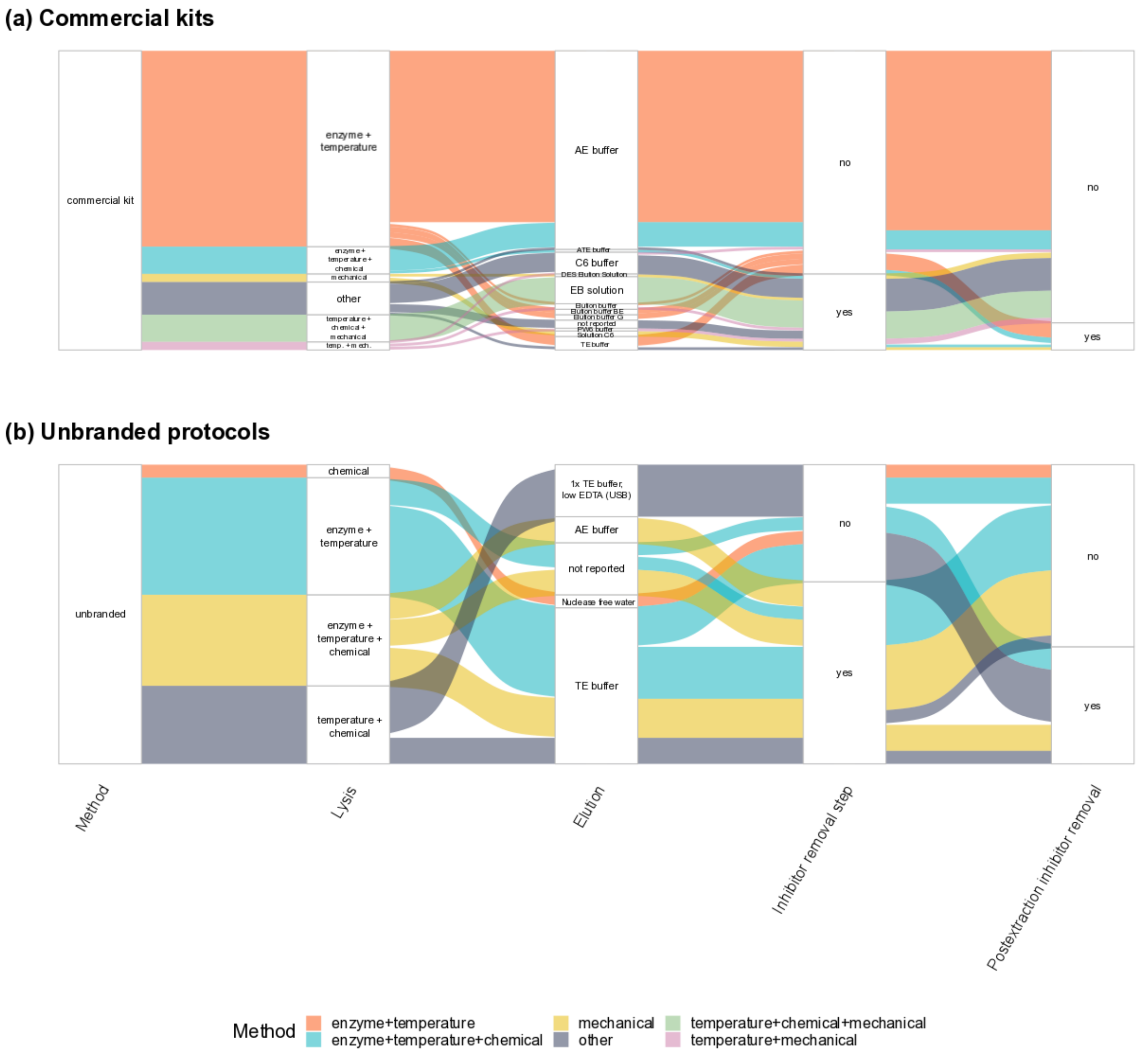

Figure S10. Sankey diagram summarising the commercial **(a)** and unbranded **(b)** extraction workflows used by different assays.

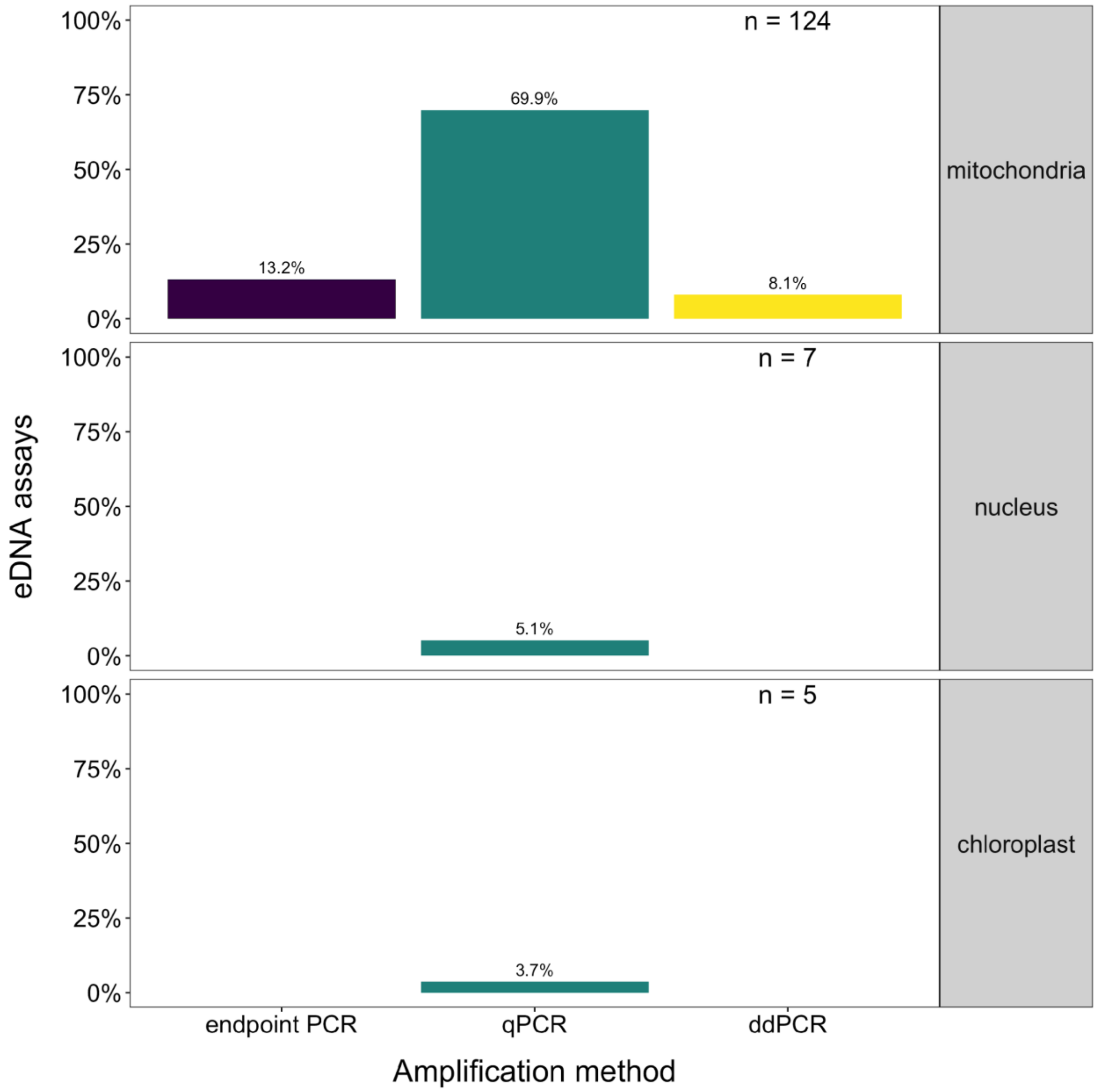

Figure S11. Bar plot summarising the amplification method and target gene used by different assays.

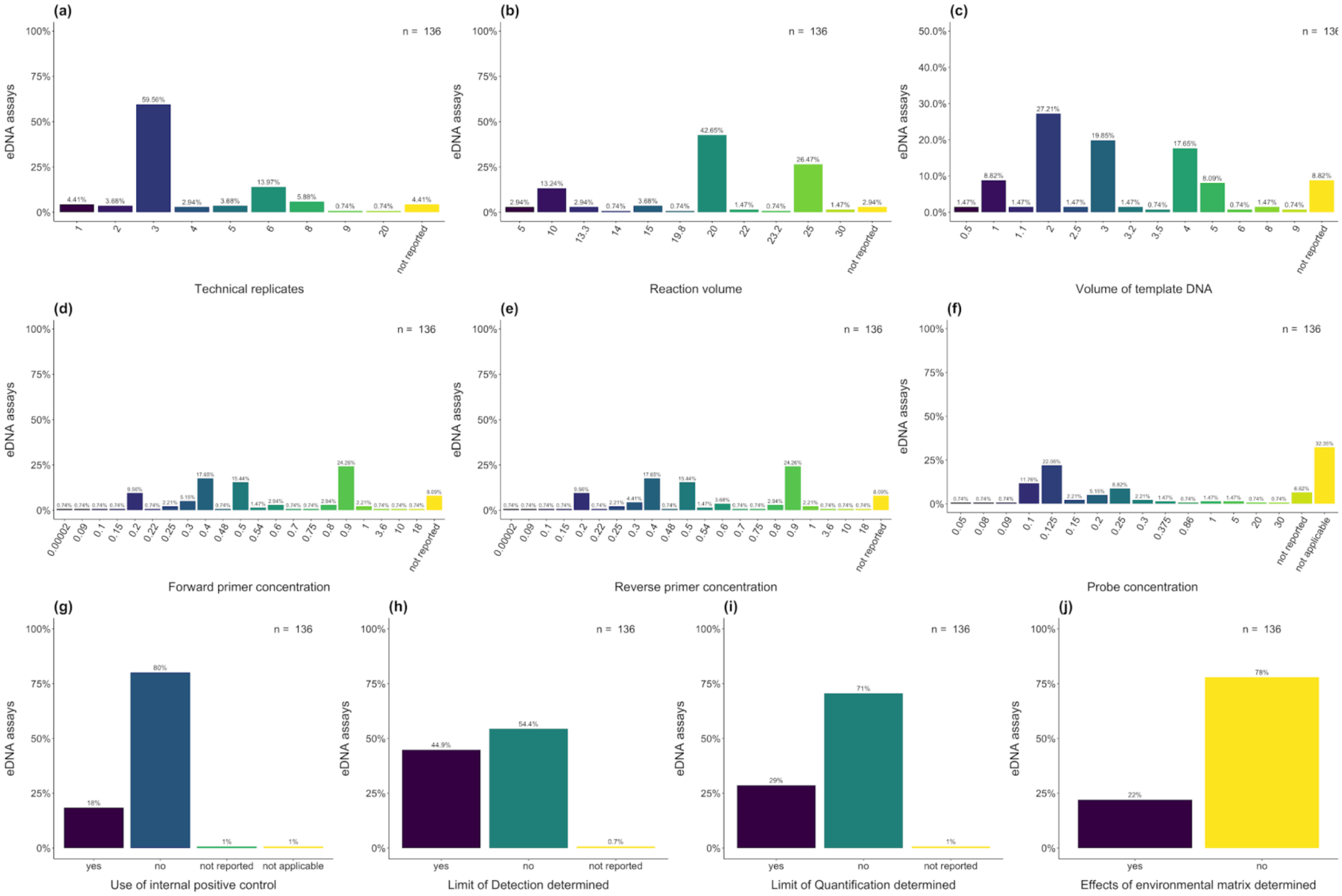

Figure S12. Bar plots summarising amplification conditions and extent of *in vitro* validation for different assays.

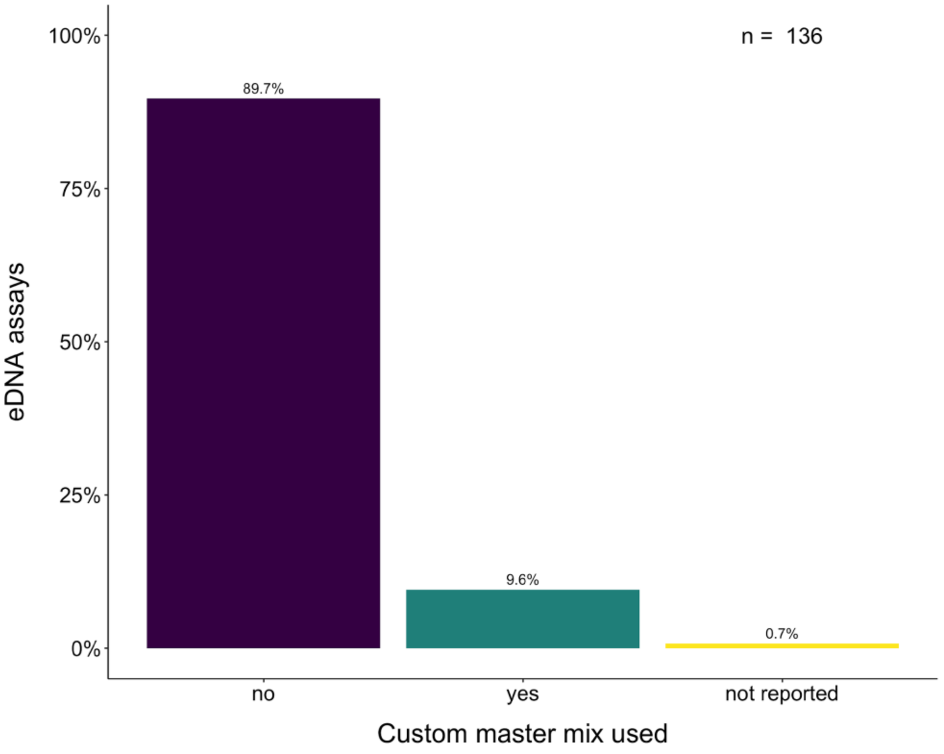

Figure S13. Bar plot summarising whether assays used a custom master mix for amplification.

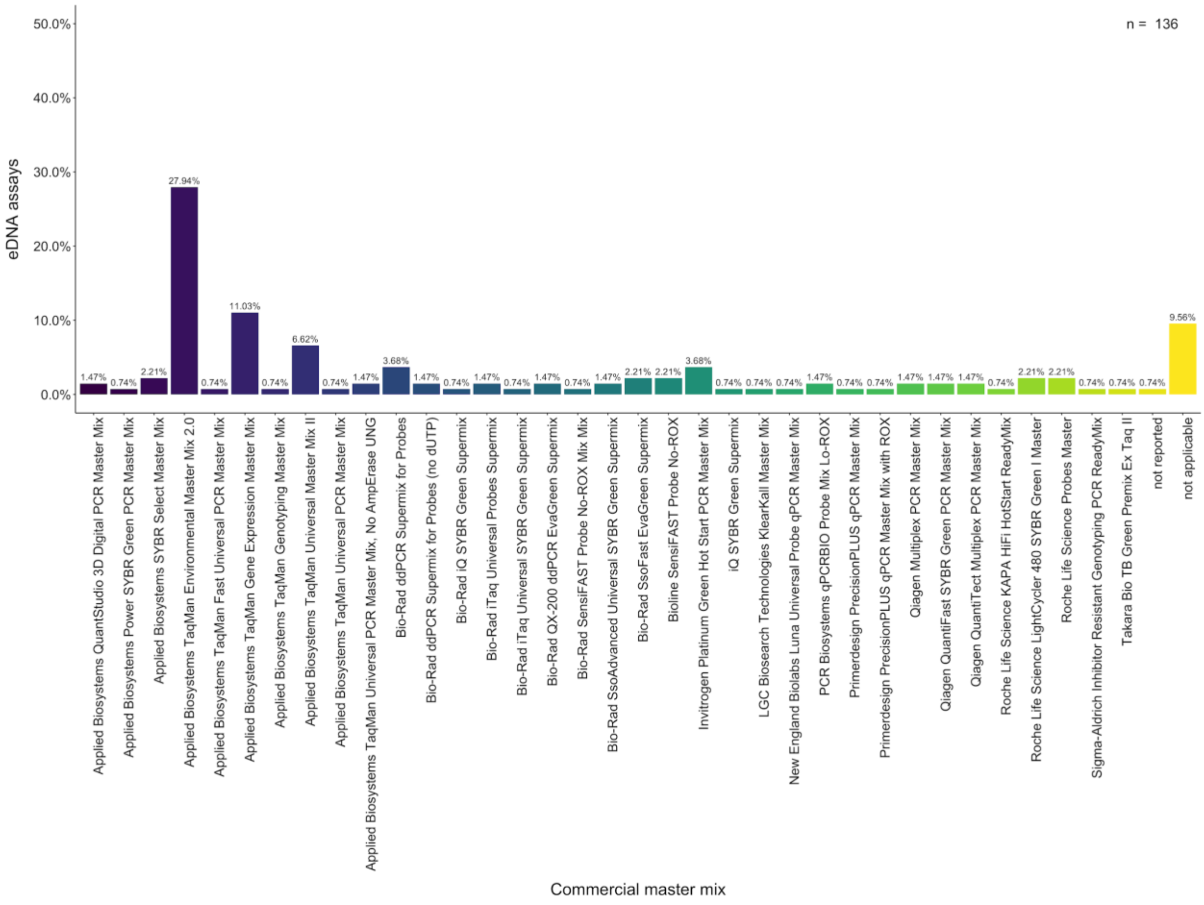

Figure S14. Bar plot summarising the commercial master mixes used by different assays.

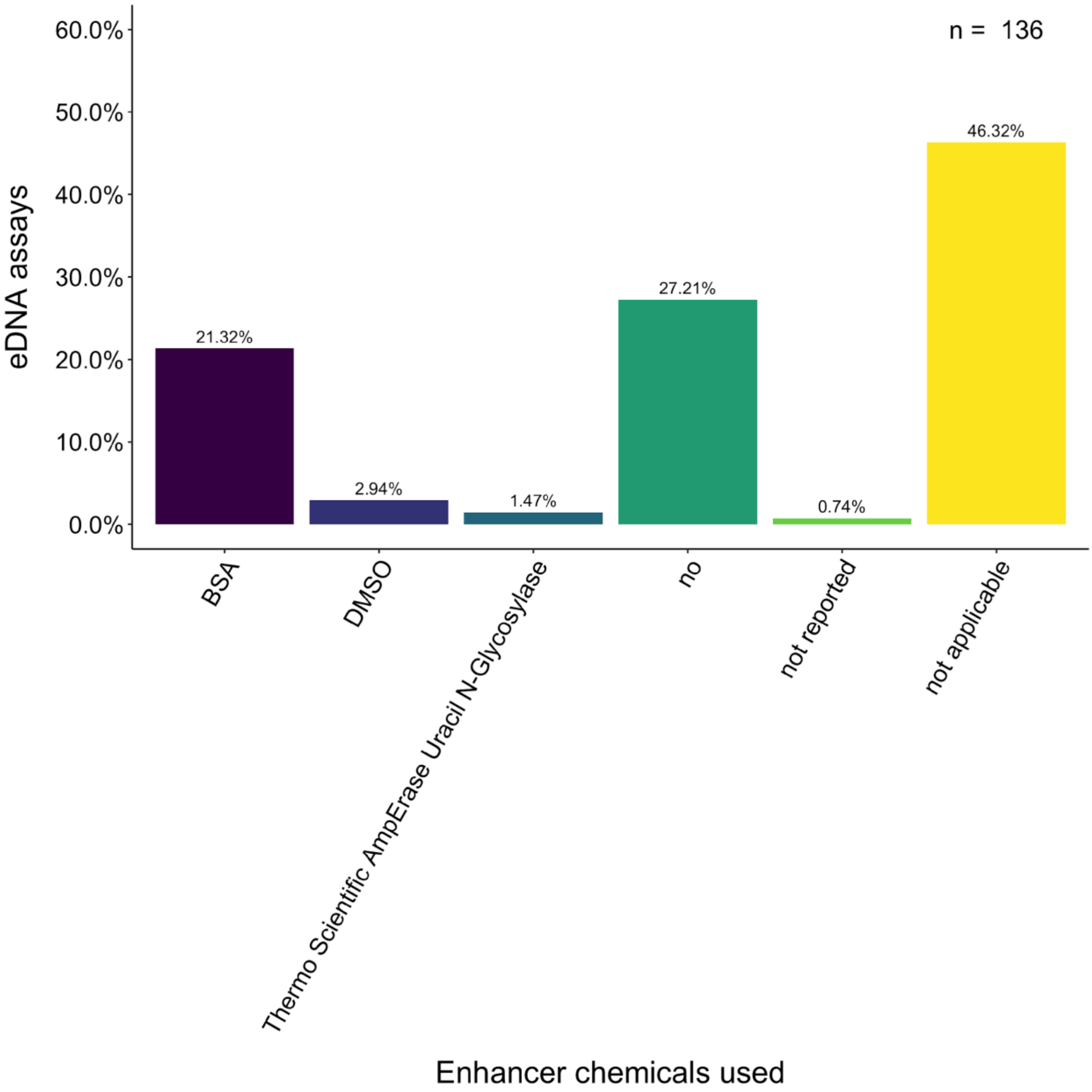

Figure S15. Bar plot summarising the number of assays using enhancer chemicals in their amplification reactions.

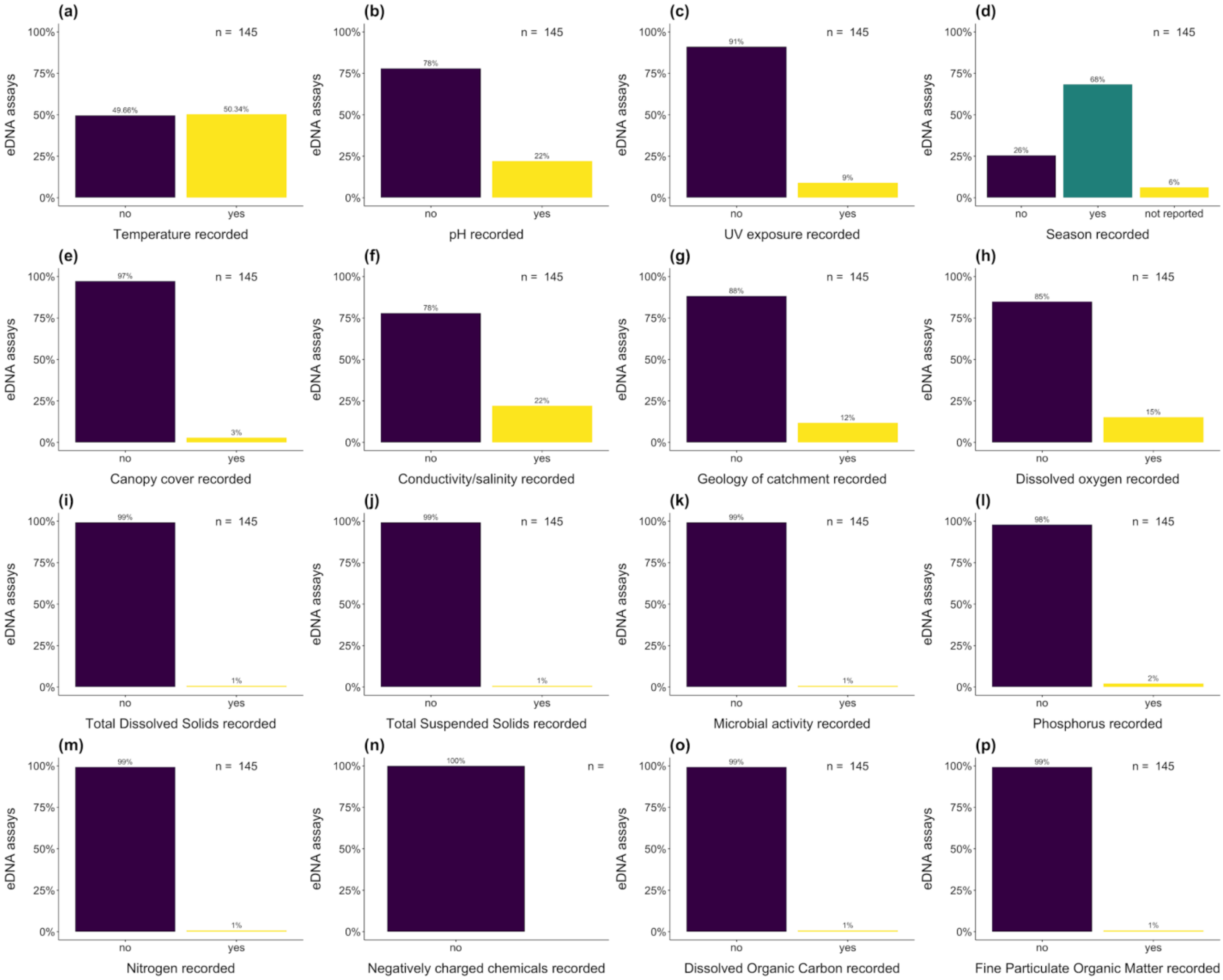

Figure S16. Bar plots summarising the number of assays reporting environmental conditions

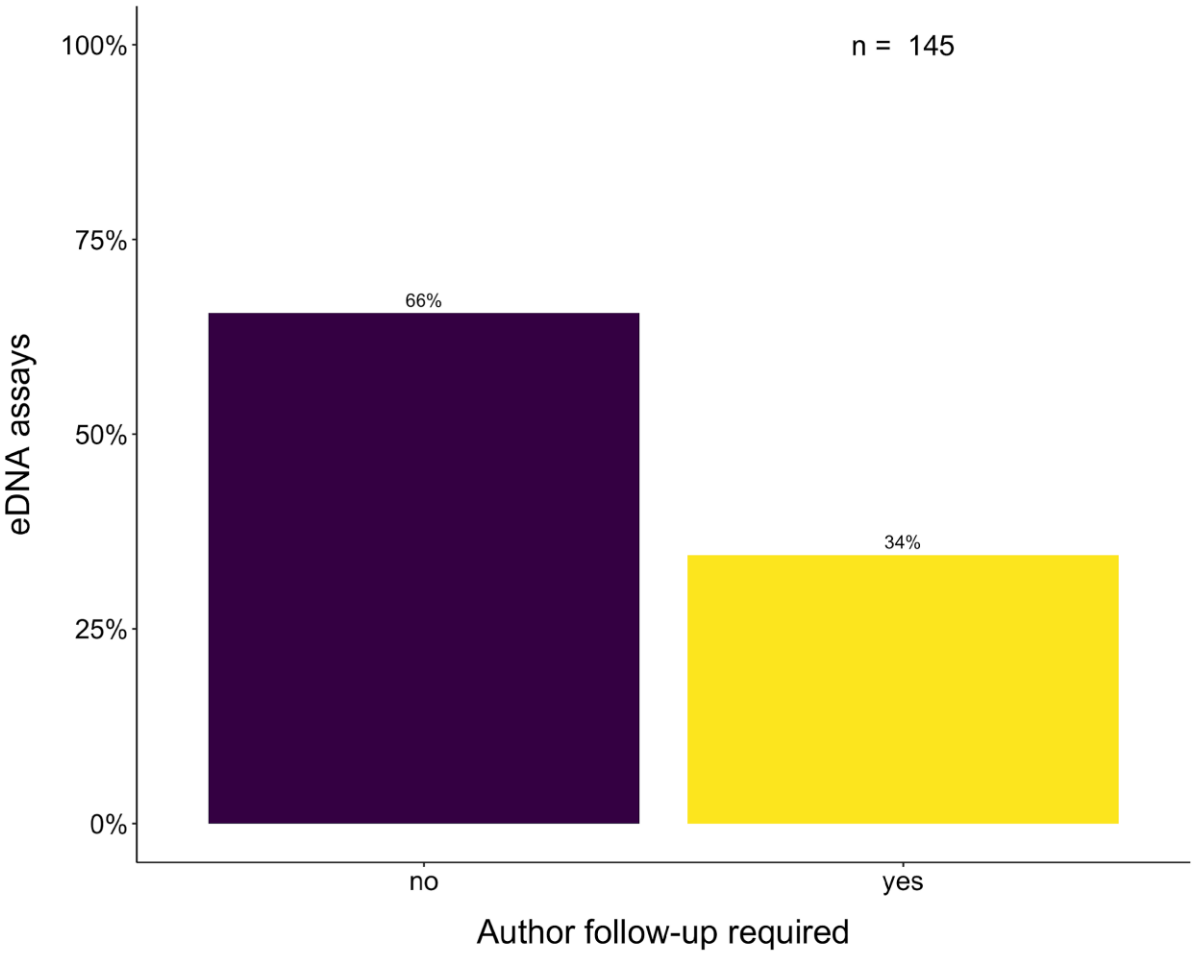

Figure S17. Bar plot summarising whether authors of a given assay would need to be contacted to clarify ambiguities in their eDNA workflow.
